# A pilot feasibility randomised controlled trial of an online cancer bereavement group

**DOI:** 10.1016/j.invent.2026.101017

**Published:** 2026-10-31

**Authors:** Lara King, Kirsten V. Smith, Charles L. Cole, Erin Hope Thompson

**Affiliations:** a Division of Psychology & Lang Sciences, Faculty of Brain Sciences, Research department of Clinical Educational and Health Psychology (CEHP), University College London (UCL), London, UK; b The Loss Foundation, [Registered Charity 1147362], London, UK; c Department of Experimental Psychology, University of Oxford, Oxford, UK; d Oxford Health NHS Foundation Trust, Oxford, UK

**Keywords:** Bereavement, Prolonged grief, Cancer, Internet intervention, Group therapy, Cognitive behavioural therapy, Pilot randomised controlled trial

## Abstract

**Background:**

Bereavement following the death of a loved one from cancer presents unique challenges related to anticipatory stressors, caregiving demands, and the illness trajectory. Although online bereavement interventions may improve access to support, evidence for therapist-led online groups remains limited. This pilot randomised controlled trial evaluated the feasibility, acceptability and preliminary effectiveness of an online therapeutic group intervention for adults bereaved by cancer.

**Methods:**

In this longitudinal pilot RCT, adults bereaved by cancer were randomised to receive the intervention immediately or following a three-month wait-list period. The intervention comprised eight online group sessions delivered over 12 weeks, integrating trauma-informed cognitive behavioural therapy, compassion-focused therapy and psychoeducation. Primary outcomes were feasibility and acceptability. Secondary outcomes included prolonged grief, depression, anxiety, post-traumatic stress, self-compassion, and social disconnection, assessed at baseline, post-intervention, and three-month follow-up.

**Results:**

Of 124 randomised participants, 100 (80.6%) commenced the allocated intervention and 97 contributed sufficient data for primary analyses. The study met pre-specified feasibility and acceptability criteria, although patterns of attrition differed according to trial stage. Non-commencement following allocation was higher in the immediate intervention groups (27.0 vs 11.5%) whereas withdrawal after intervention commencement was higher in the waitlist group (24.1% vs 10.9%). Exploratory between-group analyses suggested greater improvements in prolonged grief (d = −1.87), post-traumatic stress (d = −1.55), depression (d = −1.03), anxiety (d = −0.71), and self-compassion (d = 1.15) in the immediate intervention group compared with waitlist controls. No statistically significant between-group difference was observed for social disconnection (d = −0.49).

**Conclusions:**

Therapist-led online bereavement groups appear feasible and acceptable for adults bereaved by cancer and show promise for improving psychological outcomes. A fully powered RCT is warranted to establish efficacy.

## Highlights

• Recruitment and intervention uptake met prespecified feasibility criteria.
• Session attendance exceeded the prespecified adherence criterion.
• Retention and questionnaire completion met overall progression criteria.
• Participants reported positive facilitator and group experiences.
• Findings support a definitive trial with refinements to retention procedures.

## 1 Introduction

### 1.1 Background and rationale

Cancer is among the leading causes of death worldwide, with approximately 10 million deaths annually (Bray et al., 2024), resulting in a substantial number of cancer-bereaved individuals. Although grief is a normative response to loss, a minority develop persistent and clinically significant difficulties, including Prolonged Grief Disorder (PGD), depression, anxiety and post-traumatic stress symptoms (Mattson et al., 2025; Smith et al., 2020a). Cancer bereavement may be particularly complex because death can follow prolonged illness, anticipatory grief, caregiving demands, changing prognoses and exposure to suffering and physical deterioration (Williams and McCorkle, 2011). These experiences may contribute to grief-related distress alongside trauma-related symptoms, changes in identity and relationships, and difficulties adapting to life after loss.

Cognitive-behavioural models of prolonged grief identify processes including avoidance, maladaptive appraisals, rumination and difficulties integrating memories as mechanisms that may maintain persistent distress (Smith and Ehlers, 2021, Smith and Ehlers, 2023). Psychological interventions may therefore target these processes by supporting engagement with grief-related experiences, developing more adaptive appraisals, integrating memories of the deceased and facilitating meaning-making and adjustment. Therapeutic change may consequently involve developing greater capacity to experience and respond to grief while adapting to life following the loss, rather than eliminating grief itself.

Group interventions may offer additional mechanisms through normalisation, mutual validation, universality and peer modelling, potentially reducing social isolation and facilitating connection with others who have experienced bereavement (Smith et al., 2020b; Robinson and Pond, 2019). Therapist facilitation may further provide emotional containment, psychoeducation, structured pacing and support for engagement with difficult emotions. A therapist-led group intervention may therefore combine grief-specific therapeutic processes with interpersonal and therapist-mediated mechanisms.

Online delivery may increase access to bereavement support by reducing geographical, travel and service-availability barriers (Andriessen et al., 2019). However, digital delivery may also present challenges, including technological difficulties, digital exclusion, reduced non-verbal communication, screen fatigue and difficulties establishing group cohesion. Online interventions should therefore be evaluated not simply as substitutes for in-person care, but in relation to the therapeutic and group processes through which they are expected to operate.

Evidence regarding therapist-led online group interventions specifically for cancer bereavement remains limited. A rapid review found that online bereavement interventions incorporating therapist-guided CBT were feasible, acceptable and associated with improvements in grief, depression and stress; however, cancer-specific interventions were delivered individually rather than in groups (Finucane et al., 2025; Kaiser et al., 2022, Kaiser et al., 2023; Sveen et al., 2021). Conversely, therapist-led online group interventions have generally not focused specifically on cancer bereavement (Wagner et al., 2022; Park and Cha, 2023). Although several quality improvement projects have described cancer bereavement groups (Morris et al., 2021; Morris, 2025), evidence evaluating protocolised therapist-led online group interventions for adults bereaved by cancer remains limited beyond bereaved parents (Phillips et al., 2025).

The existing literature also provides limited insight into potential mechanisms of change. Research has predominantly examined symptom outcomes, while processes such as group cohesion, therapeutic alliance, social connectedness, grief-related cognitions, avoidance and self-compassion have been assessed less consistently. Examining a broader range of psychological outcomes may therefore provide an initial indication of whether an intervention is influencing the difficulties it was designed to address.

The Loss Foundation is a UK charity providing free support to people bereaved by cancer. Its therapist-led bereavement programme was developed within a real-world service context and incorporates CBT-informed and compassion-focused approaches alongside the therapeutic benefits of group participation. Previous research evaluating the in-person programme found increased self-compassion and reductions in grief intensity, post-traumatic stress, depression and anxiety, although social disconnection did not change (Jerome et al., 2019). A subsequent larger service evaluation (*N* = 68) replicated improvements across several psychological outcomes and suggested that changes in cognitive-behavioural processes, including memory integration, negative appraisals and maladaptive coping, may be associated with improvement (Smith et al., 2025). These findings provide a rationale for evaluating an online adaptation while examining whether its therapeutic and relational components can be delivered in this format.

The present study evaluated an online adaptation of The Loss Foundation's therapist-led bereavement group for adults bereaved by cancer. The study was primarily designed as a pilot randomised controlled trial of feasibility and acceptability, rather than a definitive test of effectiveness. Specifically, we examined recruitment, randomisation, intervention delivery, retention, attendance, participant satisfaction and safety to determine whether progression to a future fully powered trial was warranted. Preliminary changes in psychological outcomes were also examined to estimate the direction and magnitude of potential treatment effects and inform the design of a definitive evaluation.

The intervention was expected to influence grief-related distress through interconnected therapeutic, interpersonal and delivery-related pathways. Grief-specific components were expected to target avoidance, maladaptive appraisals and difficulties integrating the loss; group participation was expected to facilitate normalisation, validation and social connection; and therapist facilitation was expected to support emotional containment and structured engagement with difficult experiences. Online delivery was expected to improve accessibility while potentially introducing challenges to engagement and group cohesion. Accordingly, outcomes were assessed across grief, post-traumatic stress, depression, anxiety, self-compassion and social disconnection.

### 1.2 Study aims

1. The primary objective was to evaluate the feasibility and acceptability of delivering a therapist-led online bereavement group for cancer loss, assessed through recruitment, randomisation, retention, attendance, satisfaction and adverse outcomes.
2. The secondary aim was to explore preliminary effectiveness by examining whether the immediate intervention group improved on psychological outcomes compared to the waitlist control at post-intervention.

It was hypothesised that relative to the waitlist control, participants in the immediate intervention group would show reductions post-intervention in grief intensity, post-traumatic stress, depression and anxiety, alongside increased self-compassion, with changes maintained at follow-up 3 months later. These hypotheses were informed by the results of Jerome et al. (2019) and Smith et al. (2025) alongside previous bereavement literature indicating preliminary effectiveness of online therapist-led groups (Phillips et al., 2025; Wagner et al., 2022).

## 2 Methods

### 2.1 Design

This study employed a longitudinal pilot randomised controlled trial (RCT) design to evaluate the feasibility, acceptability and preliminary clinical outcomes of an online bereavement group for adults bereaved by cancer. The study was conducted in accordance with CONSORT 2025 guidelines and the extension for pilot and feasibility trials (Hopewell et al., 2025; Eldridge et al., 2016). Participants were randomly allocated to one of two parallel arms: an immediate intervention group (non-waitlist) or a waitlist control group, which received the same intervention following a 3-month delay. A waitlist approach was selected as an ethical and pragmatic comparator in the context of limited service capacity (Mohr et al., 2009).

Four online groups were delivered (two immediate intervention and two waitlist control groups) to maintain clinically appropriate group sizes and accommodate the waitlist design within the study timeframe. The intervention ran over 12 weeks and comprised eight sessions: four weekly sessions followed by four fortnightly sessions, allowing time for consolidation and application of learning. The waitlist control group commenced 3 months later and followed the same schedule (See Appendix B for the study timeline).

#### 2.1.1 Changes to trial protocol

No substantive changes to the trial design, eligibility criteria, outcomes or planned analyses were made following trial commencement. Minor procedural adaptations were implemented to support engagement and accessibility. For example, several participants reported not receiving study emails because messages had been filtered into spam folders. In response to this, reminder emails were supplemented with follow-up contact via telephone or text message to ensure participants received study communications and to support completion of outcome measures. Follow-up contact was also used to check in with participants who did not attend a group session without informing the facilitators or research team in advance.

### 2.2 Participants

#### 2.2.1 Patient and public involvement

The Loss Foundation routinely undertakes patient and public involvement and service-development activity, including seeking feedback from service users and facilitators following its therapeutic services. This ongoing feedback informed the development and refinement of the intervention, structure, content and delivery. However, service users were not directly involved in the design, conduct or reporting of the present trial.

#### 2.2.2 Eligibility criteria

Participants were eligible if they were adults (≥18 years) bereaved by cancer (e.g., partner, family member or friend), at least six months post-bereavement, able to access the intervention online and willing to be randomised and potentially allocated to the waitlist control condition. The six-month criterion was applied to minimise interference with natural recovery processes in grief (Stroebe and Schut, 2010).

Exclusion criteria included high risk of harm to self or others, significant substance or alcohol misuse or concurrent engagement in psychological therapy. Risk was assessed during telephone screening. Individuals identified as high risk were immediately referred to The Loss Foundation's in-house Clinical Psychologist for risk assessment and signposting to suitable support services, consistent with ethical guidance for psychological trials (Duggan et al., 2014).

#### 2.2.3 Recruitment

Participants were recruited via The Loss Foundation (TLF) through advertisements distributed on its mailing list, website and social media platforms (Facebook and Instagram) (see Appendix C). Interested individuals initially registered their interest in being contacted about the proposed study via a secure online link, providing their contact details. This initial registration constituted an expression of interest rather than formal enrolment in the trial.

Individuals were subsequently invited to a telephone screening call conducted as part of TLF's established service procedures to assess suitability for the therapeutic groups. During these calls, individuals were also informed about the proposed study, including what participation would involve should ethical approval be granted, and were given the opportunity to ask questions and express their interest in participating.

Following UCL research ethics approval on 4 February 2025, prospective participants were provided electronically with the full participant information sheet (see Appendix D), which outlined the study procedures, including randomisation and the possibility of delayed intervention as part of the waitlist design. Participants were asked to confirm by email that they were happy to proceed with participation in the trial. This confirmation was obtained prior to randomisation. Formal electronic consent was subsequently recorded alongside baseline outcome measures, following allocation and before participation in the intervention (see Appendix E).

### 2.3 Intervention

The intervention was delivered remotely via Zoom in eight group sessions (4 weekly and 4 fortnightly) of approximately two hours. The programme was developed by the second and last author and refined over more than 15 years of clinical implementation and service evaluation. Originally developed as a six-session intervention, it was expanded to eight sessions to allow greater discussion and incorporate principles of Continuing Bonds Theory, which emphasises maintaining symbolic and evolving connections with a deceased loved one (Klass et al., 1996; Wild et al., 2023). Fig. 1 provides a visual overview of the intervention structure and sequence to aid readers' understanding of the format and sequence of the online bereavement programme. The illustration was generated using OpenAI's GPT-5.5 (June 2026).

**Fig. 1:**
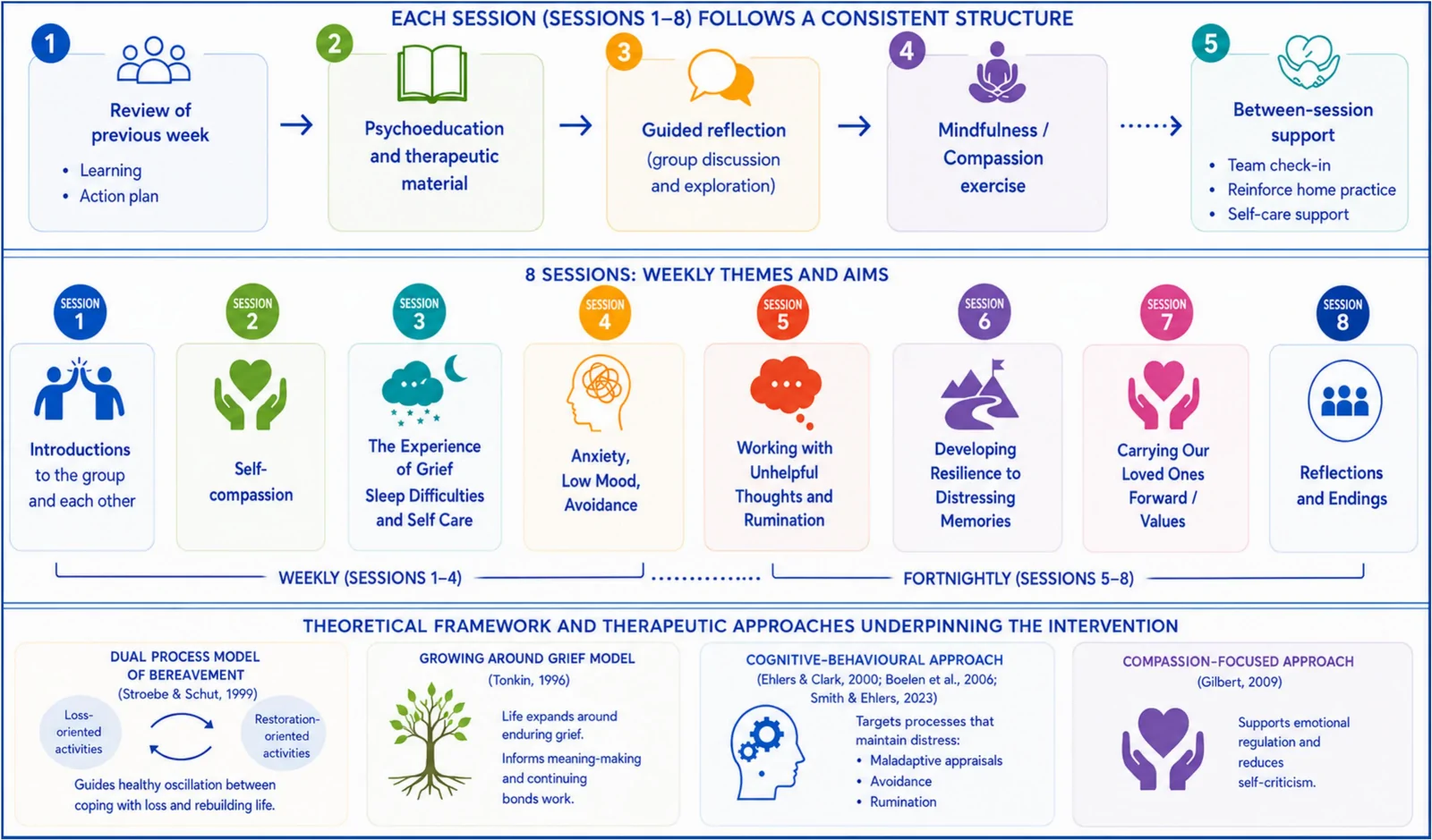
Intervention infographic, illustrating the structure of the therapist-led online cancer bereavement group. The illustration was generated using AI technology (OpenAI, GPT-5.5, June 2026) based on author-designed content and subsequently reviewed and edited by the authors.

The intervention was primarily informed by a cognitive-behavioural, transdiagnostic model of bereavement, in which persistent grief-related distress is maintained by interacting processes involving loss-related memories, maladaptive appraisals, maladaptive coping strategies (including avoidance and rumination), and social disconnection (Boelen et al., 2006; Smith and Ehlers, 2021, Smith and Ehlers, 2023). These processes overlap with mechanisms implicated in post-traumatic stress following bereavement and provided the rationale for addressing both grief and PTSD symptoms within the same intervention (Ehlers and Clark, 2000; Smith and Ehlers, 2021). Recent evaluation of the programme has also identified improvements in these cognitive-behavioural processes alongside reductions in prolonged grief, PTSD, depression and anxiety (Smith et al., 2025).

Accordingly, intervention content targeted integration of the loss into autobiographical memory, modification of maladaptive grief- and trauma-related appraisals, reduction of avoidance and rumination, adaptive behavioural engagement, self-compassion and emotional regulation. The intervention was not a trauma-focused PTSD treatment and did not include formal exposure or Cognitive Therapy for PTSD. Rather, PTSD symptoms were expected to improve indirectly through modification of cognitive and behavioural processes shared by prolonged grief and post-traumatic stress, particularly avoidance, maladaptive appraisals and difficulties processing distressing memories.

The Dual Process Model of Coping with Bereavement provided an overarching framework for sequencing the intervention, encouraging movement between loss-oriented and restoration-oriented coping (Stroebe and Schut, 1999). Continuing Bonds Theory and the Growing Around Grief model informed later work on meaning reconstruction, maintaining adaptive connections with the deceased and re-engagement with valued living (Klass et al., 1996; Tonkin, 1996). Compassion-focused principles were integrated throughout to support emotional regulation, reduce self-criticism and facilitate engagement with difficult grief-related experiences (Gilbert, 2009). These frameworks therefore informed the content and delivery of the intervention, while the cognitive-behavioural model provided the primary account of its proposed mechanisms of change.

Sessions followed a structured format comprising review of previous learning and action plans, psychoeducation and therapeutic content, guided reflection, and a mindfulness- or compassion-based exercise. Participants also received weekly email contact from the research team to reinforce engagement with home practice and self-care. Session content included:

**Week 1**: Introductions and group orientation.

**Week 2:** Self-compassion.

**Week 3:** The experience of grief, sleep difficulties and self-care.

**Week 4:** Anxiety, low mood, avoidance.

**Week 5**: Working with unhelpful thoughts and rumination.

**Week 6:** Developing resilience to distressing memories.

**Week 7**: Carrying our loved ones forward and values.

**Week 8:** Reflections and endings.

Participants allocated to the waitlist control group received the intervention after a three-month delay. During the waiting period, they received written psychoeducational materials and signposting to support services, including self-guided grief resources and information about signs of declining wellbeing (Appendix D). The three-month delay also reflected practical constraints associated with limited capacity to run groups concurrently.

#### 2.3.1 Facilitation and fidelity

Groups were co-facilitated by trained clinicians (Clinical Psychologists and Cognitive Behavioural Therapists) with experience in bereavement support (*n* = 8) alongside volunteers in psychological support roles (including research students or assistant psychologists, *n* = 4). Facilitators completed standardised training and received ongoing supervision. A manualised protocol was used to ensure consistency of delivery (see Appendix F for a section of the intervention manual). Intervention fidelity was monitored through session checklists and any deviations were recorded by facilitators and shared with the research team.

### 2.4 Measures

Outcome data were collected at baseline, after each session, post-intervention and at 3-month follow-up. Participants in the waitlist control group completed baseline measures twice (pre- and post-waiting period) to assess change during the control phase. All data were collected online via The Loss Foundation's Qualtrics platform. Secure email links and automated reminders were used to maximise response rates.

#### 2.4.1 Primary outcomes: feasibility and acceptability

Consistent with guidance for pilot trials, the primary outcomes focused on feasibility and acceptability rather than definitive effectiveness (Eldridge et al., 2016; Thabane et al., 2010). Feasibility outcomes were assessed against pre-specified progression criteria using a traffic-light framework (green, amber, red), informed by recommendations for pilot and feasibility studies (Avery et al., 2017; Appendix G).

Progression criteria were evaluated using pooled trial estimates, with arm-specific estimates examined descriptively to identify potential differences in implementation between the immediate intervention and waitlist control groups. Thus, an overall green rating indicates that the predefined pooled criterion was met, while arm-level values were used to identify areas requiring attention in a future definitive trial.

I. Recruitment and uptake: Feasibility was assessed using recruitment metrics, including referral source, expressions of interest, eligibility, consent and randomisation rates. Recruitment was considered feasible if ≥70% of the target was reached within the planned period.

II. Adherence and attrition: Session adherence was monitored via Zoom attendance records stored securely on The Loss Foundation server. Feasibility for adherence was predefined as ≥60% and data completeness was considered acceptable if ≥70% of participants provided full outcome data. Retention strategies included emphasising attendance expectations at screening, fostering a supportive and reflective group environment, encouraging between-session practice tasks and sending follow-up reminders.

III. Acceptability: Acceptability was assessed using the Helpful Aspects of Therapy (HAT) questionnaire after each session, capturing perceived helpful and unhelpful aspects (Llewelyn, 1988). At intervention completion, participants also rated session length, online delivery, facilitator effectiveness and group size, using unvalidated Likert-scale items. Acceptability thresholds were predefined as ≥70% of participants attending at least 50% of sessions and reporting positive ratings of session usefulness and format. HAT feedback was summarised and shared with facilitators before each session to inform ongoing therapeutic delivery. Safety and adverse events were monitored throughout the intervention period.

#### 2.4.2 Secondary outcomes: psychological outcomes

Secondary outcomes comprised routinely collected psychological measures used by The Loss Foundation. Measures were selected based on previous evidence of sensitivity to change and alignment with transdiagnostic intervention models. Weekly outcome measures were used to examine change trajectories over time. These outcomes were exploratory and helped to inform outcome selection and sample size estimation for a future definitive trial (Leon et al., 2011; Thabane et al., 2010).

#### 2.4.3 Weekly pre and post-session measures

Depressive symptoms were assessed using the Patient Health Questionnaire-9 (PHQ-9; Kroenke et al., 2001), anxiety using the Generalised Anxiety Disorder-7 (GAD-7; Spitzer et al., 2006), grief symptoms using the Prolonged Grief Disorder-13-Revised (PG-13-R; Prigerson et al., 2009, Prigerson et al., 2021) and post-traumatic stress symptoms using the PTSD Checklist for DSM-5 (PCL-5; Weathers et al., 2013). Social disconnection was assessed using the Oxford Grief-Social Disconnection Scale (OG-SD; Smith et al., 2020b) and self-compassion using the Self-Compassion Scale (SCS; Raes et al., 2011). The Helpful Aspects of Therapy (HAT; Llewelyn, 1988) was used to capture perceived helpful and unhelpful aspects of the intervention. All measures demonstrate good psychometric properties in general populations (see Appendix H for details). Although widely used in bereavement research, these measures have not all been specifically validated in bereaved populations.

#### 2.4.4 Demographics and bereavement characteristics

The Loss Foundation routinely collects participant demographics (age, gender, ethnicity, education, employment and prior psychological treatment) and bereavement characteristics (relationship to the deceased, cohabitation status, contact prior to death, presence at death and recent losses). Data were securely stored and considered sensitive under the General Data Protection Regulation but reported to evaluate service access and potential barriers.

#### 2.4.5 Harms

The criterion for discontinuing the intervention was defined as any adverse outcomes involving sustained deterioration directly caused by the psychological intervention (Duggan et al., 2014). Adverse outcomes were monitored weekly via self-report and via item-9 on the PHQ-9 which assesses thoughts of death or self-harm. Scores of two or three on this item triggered automated alerts in Qualtrics and were reviewed by the research team. While this threshold did not indicate the need for immediate intervention necessarily, scores were reviewed over the course of the group. If elevated scores were sustained by the end of the intervention, the research team sent an email check-in with the participant. These actions were in accordance with the study's predefined risk management protocol.

### 2.5 Randomisation, allocation and blinding

Following confirmation of eligibility and documented agreement to participate, participants were randomly allocated in a 1:1 ratio to either the immediate intervention or waitlist control group using a secure web-based randomisation system (Urbaniak and Plous, 2013). Randomisation commenced on 7 February 2025, following ethical approval granted by the Life and Medical Sciences Research Ethics Committee (LMS REC) at University College London (UCL) on 4 February 2025 (Project ID No: 0183). Group allocation was communicated to participants by email. Following allocation, participants completed the electronic consent form (see Appendix E) alongside their baseline outcome measures, before commencing the intervention.

Due to the nature of the intervention, neither participants nor facilitators were blinded to allocation. Participants were therefore aware of their allocation when completing baseline measures. As simple randomisation can result in unequal group sizes or baseline imbalances in small samples, these were examined descriptively and considered when interpreting exploratory outcomes (Schulz and Grimes, 2002).

### 2.6 Sample size

A total of 124 adults bereaved by cancer were randomised (immediate intervention *n* = 63, waitlist *n* = 61), with 100 commencing the intervention. As a pilot RCT, the sample size was determined to assess feasibility outcomes, rather than to provide definitive evaluation of clinical effectiveness or achieve formal statistical power (Eldridge et al., 2016; Teare et al., 2014; Totton et al., 2023). No interim analyses were conducted and no formal stopping guidelines were applied. Based on previous research (Smith et al., 2025), a between-group effect size of *d* = 0.6 informed an a priori sample size of approximately 100 participants (50 per arm), estimated using G*Power (Faul et al., 2007).

The trial was designed to recruit 100 adults bereaved by cancer, with 50 participants in each arm. A total of 124 participants were ultimately randomised: 63 to the immediate intervention group and 61 to the waitlist control group. As a pilot RCT, the target sample was selected to estimate key feasibility parameters, including recruitment, consent, randomisation, adherence, retention, acceptability and data completeness, with acceptable precision rather than to provide a definitive evaluation of clinical effectiveness (Eldridge et al., 2016; Teare et al., 2014; Totton et al., 2023). Although the study was not powered for formal efficacy testing, it enabled exploratory estimation of between-group differences. Clinical outcome estimates were interpreted cautiously and will inform outcome selection, variance estimates and sample-size planning for a future definitive RCT.

Consistent with pilot trial methodology, analyses of clinical outcomes were exploratory and intended to generate preliminary effect size estimates to inform the design and sample size calculation of a future definitive trial. The target sample size also reflected anticipated attrition and the practical requirements of delivering group-based intervention sessions. Online therapeutic groups were structured for 25 participants per group to ensure engagement, safety and effective facilitation.

### 2.7 Statistical analyses

Analyses were conducted using JASP (JASP Team, 2025, Version 0.19.3). Data were screened for outliers, missingness and normality. Residual distributions, homogeneity of variance and model fit assumptions were assessed and found to be acceptable, therefore, non-parametric alternative analyses were not required.

#### 2.7.1 Primary analysis (feasibility outcomes)

Feasibility and acceptability outcomes including recruitment, consent, retention, adherence, data completeness and helpfulness of the intervention were summarised descriptively and compared against predefined Progression criteria (see Appendix G).

#### 2.7.2 Secondary exploratory analysis (clinical outcomes)

Outcome data were defined a priori as assessments collected at baseline, post-intervention and 3-month follow-up. Weekly sessional outcome measures (excluding social disconnection) were treated as supplementary exploratory data and were not included in the primary outcome analyses. Clinical outcomes were analysed using an available-data approach. The primary analysis population comprised 97 randomised participants with available baseline outcome data. Participants with missing data were excluded only from analyses requiring those observations; consequently, the N reported for each analysis reflects the participants contributing data to the specific outcome and variables included in that model.

Linear mixed-effects models were used as the primary analytic approach, with fixed effects for group, time and their interaction and random intercepts for participants. These models incorporated all available outcome data across assessment time points and estimated treatment effects using the Group × Time interaction. Estimated marginal means and model-derived contrasts were used to examine between-group differences in change over time. Effect sizes and 95% confidence intervals were reported for all comparisons. Cohen's d values of 0.2, 0.5 and 0.8 were interpreted as small, medium and large effects (Cohen, 1988).

Missing outcome data were handled within the mixed-effects modelling framework using likelihood-based estimation, which provides valid estimates under a missing-at-random (MAR) assumption. This approach was preferred over last observation carried forward (LOCF), as likelihood-based mixed-effects models make use of all available observations without requiring the strong assumptions associated with LOCF (Verbeke and Molenberghs, 2000).

As a secondary sensitivity analysis, baseline-adjusted analyses of covariance (ANCOVA) were conducted among participants with available post-intervention data. These models compared post-treatment outcomes between groups while adjusting for baseline scores to assess the robustness of findings obtained from the mixed-effects models.

Exploratory repeated-measures ANOVA was used for weekly sessional outcomes; these analyses were limited to complete cases and interpreted cautiously due to potential bias from missing data. Reliable change indices were calculated for key outcomes between baseline, post-intervention and 3-month follow-up to estimate individual-level change. Reliable change indices were calculated using Cronbach's alpha as the reliability estimate (Jacobson and Truax, 1991). Exploratory linear mixed-effects models examined whether demographic and bereavement-related variables were associated with changes in outcomes over time (pre-post) for both groups. Models included random intercepts for participants and were estimated using restricted maximum likelihood (REML).

#### 2.7.3 Probable diagnostic status

A post hoc exploratory analysis was conducted to estimate the proportion of participants exceeding established self-report screening thresholds for Prolonged Grief Disorder (PGD) and post-traumatic stress disorder (PTSD) at baseline. Published total-score thresholds were applied to the PG-13-R (≥30; Prigerson et al., 2021 and PCL-5 (≥33; Weathers et al., 2013), respectively, rather than item-level diagnostic algorithms. These thresholds were used to describe baseline symptom burden and identify participants screening positive for probable PGD or PTSD, rather than to establish clinician-confirmed diagnoses. The analysis was descriptive and was not used as an outcome measure or to determine treatment eligibility.

#### 2.7.4 Participant characteristics

Demographic and bereavement-related characteristics were summarised descriptively for the overall sample and by group, using means and standard deviations (i.e. for continuous variables and counts) and percentages for categorical variables. These data provided context for feasibility and acceptability outcomes and were used descriptively to explore potential covariates or associations with outcomes. Such analyses were descriptive and hypothesis-generating only (Eldridge et al., 2016).

## 3 Results

Feasibility and acceptability outcomes are presented first, followed by preliminary effectiveness analyses. The feasibility and acceptability of the online bereavement group were evaluated using recruitment, randomisation, retention, attendance, satisfaction and adverse outcomes. Preliminary effectiveness was examined through changes in psychological outcomes between the immediate intervention and waitlist control groups.

### 3.1 Participant flow

A total of 241 individuals expressed interest in the study, of whom 171 were assessed for eligibility. Of these, 124 met the inclusion criteria, provided consent and were randomised to the immediate intervention group (*n* = 63) or waitlist control group (*n* = 61), representing 72.5% of those assessed. Overall, 100 participants (80.6%) commenced their allocated intervention (46 immediate intervention; 54 waitlist control), while 24 (19.4%) did not initiate treatment.

Among participants commencing treatment, 18 withdrew prior to completion (5/46 immediate intervention; 13/54 waitlist control). Baseline questionnaires were returned by 97 participants (44 immediate intervention; 53 waitlist control).

At the controlled-phase endpoint, questionnaires were returned by 79 participants: 31 in the immediate intervention group following treatment and 48 in the waitlist control group following the waiting period, before receiving the intervention. Following delayed intervention delivery, post-intervention questionnaires were returned by 35 waitlist participants. Thus, post-intervention questionnaires were returned by 66 participants overall (31 immediate intervention; 35 waitlist control). At three-month post-intervention follow-up, outcome data were available for 62 participants (31 in each group).

Recruitment was completed between January and February 2025, as planned, with no modifications to trial procedures. Reasons for non-initiation, withdrawal and missing outcome data are presented in Fig. 2.

**Fig. 2:**
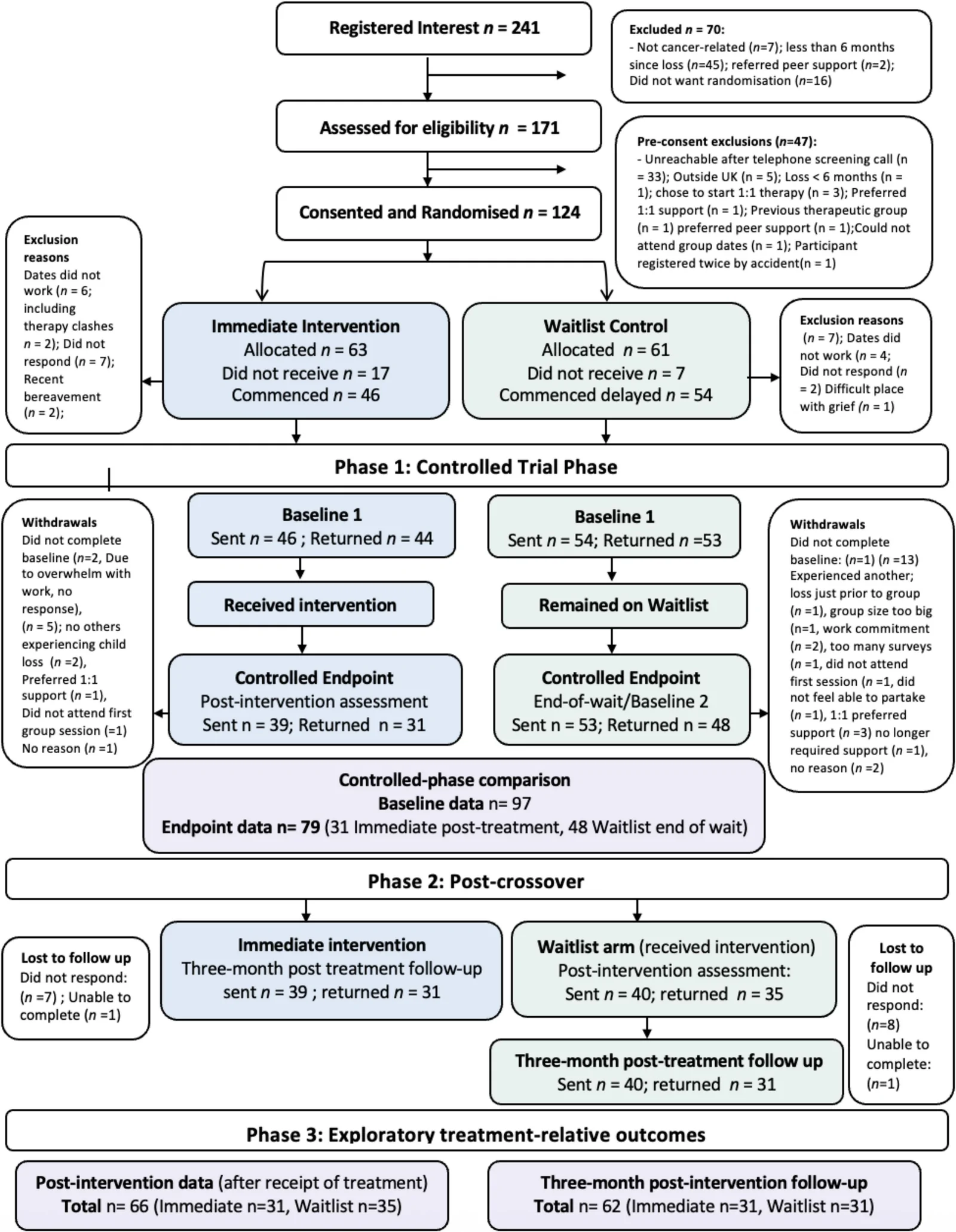
Participant flow diagram (Eldridge et al., 2016). *Note*. Non-WL = Non-waitlist Immediate Intervention Group, WL = Waitlist control groups, ET = End of treatment, 3-month follow up = follow up assessment 3 months after the end of treatment.

### 3.2 Primary outcomes

Feasibility outcomes were assessed against the pre-specified progression criteria (Table 1). Traffic-light ratings were determined using pooled trial estimates, while arm-specific estimates were examined descriptively to identify potential differences in implementation between the immediate intervention and waitlist control groups.

**Table 1:** Feasibility outcomes.

Feasibility domain	Outcome / metric	Immediate Intervention	Waitlist control group	Pooled	*Pre-specified* progression criterion	*Progression rating and interpretation*
**Recruitment & uptake**	Expressions of interest (pre-randomisation) (*N)*	–	–	241	≥70% of target	□ Criterion met; recruitment interest supported feasibility
	Eligible and consented (pre-randomisation) (%)	–	–	124 / 171 (72.5%)	≥70% eligible	□ Criterion met; Eligible/Consent acceptable
	Randomised (N, %)	63	61	124 (100%)	≥70% of target	□ Criterion met; Randomisation procedures feasible
	Did not receive allocated intervention (%)	17 / 63 (27.0%)	7 / 61 (11.5%)	24 / 124 (19.4%)	<20%	□ Overall criterion met; arm-level non-receipt exceeded the threshold in the immediate intervention group (27.0%) and should be addressed before a definitive trial.
	Withdrawals after intervention started (n/%)	5 / 46 (10.9%)	13 / 54 (24.1%)	18 / 100 (18.0%)	<20%	□ Overall criterion met; arm-level withdrawal exceeded the threshold in waitlist control group (24.1%) and should be addressed before a definitive trial.
**Retention / adherence**	Commenced intervention (n/%)	46/63 (73.0%)	54/61 (88.5%)	100/124 (80.6%)	≥70%	□ Criterion met; High uptake following allocation
	Participants attending ≥4/8 sessions, n/N (%)	42/46 (91.3%)	40/54 = 74.1%	82/100 (82.0%)	≥60%	□ Criterion met
	Means sessions attended (8 total 8) *M/SD*	6.02 (SD = 2.43)	5.31 (SD = 2.82)		≥60% attend ≥50% sessions	□ Adequate engagement across arms
**Data completeness**	Baseline questionnaire completion (n/%)	44 / 46 (95.7%)	53 / 54 (98.1%)	97 / 100 (97.0%)	≥70%	□ Criterion met; Strong baseline data capture
	End-of-treatment questionnaires returned, among those sent (n/%)	31 / 39 (79.5%)	35 / 40 (87.5%)	66 / 79 (83.5%)	≥70%	□ Criterion met; Follow-up procedures acceptable
	Three-month questionnaires returned (n/%)	30 / 39 (76.9%)	31 / 40 (77.5%)	62 / 79 (78.5%)	≥70%	□ Criterion met; Retention acceptable
	End-of-treatment outcomes available, among all randomised	31/63 (49.2%)	35/61 (57.4%)	66/124 (53.2%)		
	Three-month outcomes available, among all randomised	31/63 (47.6%)	31/61 (50.8%)	62/124 (49.2%)		
**Acceptability**	Session helpfulness (HAT3): positive ratings ≥3/5, (Total ratings across 8 sessions N (%),weekly mean range	200/235 (85.1%)3.10–3.36	190/235 (80.9%) 3.00–3.35	390/470 (83.0%) 3.00–3.36	≥3.0 for ≥50%	□ Sessions perceived as highly helpful
	≥1 additional helpful event reported (%; HAT6)	49.4%	42.4%		≥80%	□ Less than 60%
	Facilitator evaluation: learning effectiveness, safe/supportive environment, respect/empathy, flexibility and management of group dynamics *M range*	4.59–4.90	4.56–4.85		No threshold given	□ Descriptive; positive facilitator ratings
	Group process (engagement, connection, comfort expressing self), *M range*	3.54–3.90	3.59–3.71		No threshold given	□ Descriptive; positive group-process ratings
	Online preference (%)	Online (21.43%), In person (17.86%), No preference (42.86%), Hybrid (17.86%)	Online (35.29%), In person (17.65%), No preference (35.29%), Hybrid (11.76%)		No threshold given	□ Mixed preferences with minor online preference
	Serious adverse events*, N*	0	0	0	None	□ No serious adverse events
**Safety**	Unhelpful experiences (average over 8 sessions), *M* / range	13.4%; Range = 3.2–23.3%	14.0%; Range = 3.7–33.3%		No serious adverse events	□ Descriptive; unhelpful experiences were reported and monitored

**Note.** Traffic-light ratings were determined using pooled trial estimates in accordance with the pre-specified progression criteria. Arm-specific estimates are presented descriptively and were not used to determine the traffic-light rating. Where an arm-specific estimate exceeded a progression threshold despite the pooled criterion being met, this is identified in the interpretation column as an implementation issue relevant to a future definitive trial.

#### 3.2.1 Feasibility

Most pre-specified progression criteria were met, although one acceptability indicator fell within the red category (Table 1). Recruitment targets were achieved, with 72.5% of individuals assessed for eligibility subsequently consenting and being randomised. Intervention uptake following allocation was 80.6% overall, with 73.0% of participants in the immediate intervention group and 88.5% in the waitlist control group commencing treatment.

The pooled non-receipt rate was 19.4%, although non-receipt was higher in the immediate intervention group (27.0% versus 11.5%). Conversely, withdrawal following treatment commencement was higher in the waitlist control group (24.1% versus 10.9%), despite the pooled withdrawal rate of 18.0%. These differences highlight distinct challenges relating to intervention uptake and retention.

Among participants commencing treatment, 82.0% attended at least four of the eight sessions, exceeding the predefined adherence criterion of ≥60%. Participants attended a mean of 6.02 sessions (SD = 2.43) in the immediate intervention group and 5.31 sessions (SD = 2.82) in the waitlist control group. Questionnaire completion rates were 97.0% at baseline and 83.5% following intervention completion. At three-month post-intervention follow-up, outcome data were available for 62 participants.

Session helpfulness met the predefined acceptability criterion, with 83.0% of HAT3 ratings meeting the positive-rating threshold. However, the proportion reporting at least one additional helpful event fell below the predefined threshold of 60% in both groups (49.4% immediate intervention; 42.4% waitlist control). No serious adverse events were reported. Unhelpful experiences were reported in both groups and monitored throughout the intervention.

#### 3.2.2 Acceptability

Participants reported high satisfaction with the intervention and felt well supported by facilitators when reflecting on their grief. Facilitator effectiveness, (e.g., creating a safe and supportive environment, demonstrating empathy and managing group processes), were rated highly. Group process indicators (e.g., engagement, connection to others, comfort expressing oneself) were also rated positively. Session feedback indicated active therapeutic engagement, with participants identifying additional helpful experiences. Preferences for the online delivery format were mixed. Findings were broadly consistent across immediate intervention and waitlist control groups, indicating overall acceptability of the intervention.

#### 3.2.3 Harms

Reports of unhelpful events on the HAT were recorded by participants in both groups. In the immediate intervention group, weekly rates were 23.3% (Week 1), 19.5% (Week 2), 8.8% (Week 3), 6.1% (Week 4), 15.2% (Week 5), 3.2% (Week 6), 10.3% (Week 7), and 21.2% (Week 8), averaging 13.4% overall. In the waitlist control group, rates were 33.3%, 20.0%, 16.7%, 10.8%, 8.8%, 3.7%, 9.4%, and 9.1% across eight weeks, averaging 14.0% overall. For sample sizes of responses, please see Table S3 in supplementary information. Reports were unevenly distributed, with a small subgroup accounting for multiple reports across sessions. No clear descriptive pattern was identified between reporting unhelpful events and subsequent attrition. Qualitative feedback indicated that unhelpful experiences often related to group dynamics, emotional intensity, challenges engaging with therapeutic exercises and technical issues. Feedback relating to emotional exposure and engagement with online therapeutic content were outlined during the screening calls, although group size was not explicitly emphasised.

### 3.3 Baseline data

Baseline characteristics of participants who completed baseline assessments (*n* = 97; immediate intervention: *n* = 44; waitlist control: *n* = 53) are presented in Table 2. Participants had a mean age of 47 years (*SD* = 13.25) and the majority were female (89.7%). Most participants reported university-level education (73%) and prior experience of psychological support (71.1%). Participants had been bereaved for an average of three years, with the majority reporting multiple losses (69.1%). Losses most commonly involved a parent (52.6%) or partner/spouse (33%). Baseline demographic and bereavement characteristics were broadly comparable between groups, although some descriptive differences were observed, including a higher proportion of participants reporting previous psychological support in the waitlist control group; no statistically significant differences were observed (all *p* > .05; all effect sizes small).

**Table 2:** Baseline characteristics.

Characteristic	Immediate intervention (*n* = 44)	Waitlist control (*n* = 53)	Total (*N* = 97)
Age, years, *M* (*SD*)	47.67 (*13.9)*	46.4 (*12.8*)	47.0 (*13.25*)
**Gender, *n* (%)**			
Female	42 (95.5%)	45 (84.9%)	87 (89.7%)
Male	2 (4.5%)	5 (9.4%)	7 (7.2%)
Non-binary	0 (0.0%)	2 (3.8%)	2 (2.1%)
Missing/other	0 (0.0%)	1 (1.9%)	1 (1.0%)
**Education, *n* (%)**			
GCSE/secondary	1 (2.3%)	3 (5.7%)	4 (4.1%)
A-levels	3 (6.8%)	3 (5.7%)	6 (6.2%)
NVQ/BTEC	4 (9.1%)	2 (3.8%)	6 (6.2%)
Professional qualification	3 (6.8%)	3 (5.7%)	6 (6.2%)
University degree	20 (45.5%)	17 (32.1%)	37 (38.1%)
Postgraduate degree	13 (29.5%)	21 (39.6%)	34 (35.1%)
No qualifications	0 (0.0%)	2 (3.8%)	2 (2.1%)
Missing	0 (0.0%)	2 (3.8%)	2 (2.1%)
**Employment, *n* (%)**			
Employed full-time	19 (43.2%)	16 (30.2%)	35 (36.1%)
Employed part-time	9 (20.5%)	11 (20.8%)	20 (20.6%)
Self-employed	1 (2.3%)	0 (0%)	1 (1.0%)
Freelance	1 (2.3%)	0 (0%)	1 (1.0%)
Unemployed seeking work	5 (11.4%)	2 (3.8%)	7 (7.2%)
Not employed, not seeking work	2 (4.5%)	6 (11.3%)	8 (8.2%)
Student full-time	0 (0.0%)	5 (9.4%)	5 (5.2%)
Retired	4 (9.1%)	0 (0%)	4 (4.1%)
Sick leave	0 (0.0%)	1 (1.9%)	1 (1.0%)
Other	2 (4.5%)	10 (18.9%)	12 (12.4%)
Missing	1 (2.3%)	2 (3.8%)	3 (3.1%)
**Previous therapeutic support**	Yes: 26 (59.1%); No: 17 (38.6%); Missing: 1 (2.3%)	Yes: 43 (81.1%); No: 10 (18.9%) Missing 0 (0.0%)	Yes: 69 (71.1%); No: 27 (27.8%) Missing 1 (1.0%)
**Loss-related characteristics, *n* (%)**			
Time known (years), *M* (*SD*)	30.7 (*16.3*)	27.4 (*15.8*)	28.9 (*16.1*)
Time since death (years), *M* (*SD*)	3.2 (*4.9*)	3.02 (*3.1)*	3.10 (*4.01)*
Multiple losses	0 = 14 (31.8%); 1 = 14 (31.8%); 2 = 9 (20.5%); 3 = 3 (6.8%); 4 = 1 (2.3%); 5 = 1 (2.3%); Missing = 2 (4.5%)	0 = 14 (26.4%); 1 = 23 (43.4%); 2 = 12 (22.6%); 3 = 4 (7.5%) 4& 5 = 0.0%	No = 28 (28.9%); Yes = 67 (69.1%), N/A = 2 (2/1%)
Lived with deceased	Yes = 23 (52.3%); No = 19 (43.2%); Missing = 2 (4.5%)	Yes = 27 (50.9%); No = 26 (49.1%) Missing = 0 (0.0%)	Yes = 50 (51.5%); No = 45 (46.4%) Missing = 2 (2.1%)
Present at death	Yes = 29 (65.9%); No = 14 (31.8%); Missing = 1 (2.3%)	Yes = 34 (64.2%); No = 19 (35.8%), Missing 0 (0.0%)	Yes 63 (64.9%) No 33 (34.0%), Missing 1 (1.0%)
**Relationship to the deceased, *n* (%)**			
Partner/spouse	13 (29.5%)	19 (35.8%)	32 (33.0%)
Parent	25 (56.8%)	26 (49.1%)	51 (52.6%)
Child	2 (4.5%)	2 (3.8%)	4 (4.1%)
Sibling	3 (6.8%)	6 (11.3%)	9 (9.3%)
Grandparent	1 (2.3%)	0 (0.0%)	1 (1.0%)
**Ethnicity, *n* (%)**			
White British/UK	26 (59.1%)	29 (54.7%)	55 (57%)
Other White background	7 (15.9%)	12 (22.6%)	19 (20%)
Asian background	2 (4.5%)	3 (5.7%)	5 (5%)
Black background	4 (9.1%)	5 (9.4%)	9 (9%)
Mixed/other	4 (9.1%)	1 (1.9%)	5 (5%)
Prefer not to say	0 (0.0%)	1 (1.9%)	1 (1%)
Another ethnic group	1 (2.3%)	2 (3.8%)	3 (3%)
**Religion**			
Christian	14 (31.8%)	18 (34.0%)	32 (33%)
Hindu	0 (0.0%)	2 (3.8%)	2 (2.1%)
Other	0 (0.0%)	1 (1.9%)	1 (1.0%)
No religion	20 (45.5%)	20 (37.7%)	40 (41.2%)
Spiritual	9 (20.5%)	10 (18.9%)	19 (19.6%)
No religion given	1 (2.3%)	2 (3.8%)	3 (3.1%)
Sessions attended, *M* (*SD*)	6.02 (SD = 2.43)	5.31 (SD = 2.82)	

Note. Baseline characteristics are presented for participants who completed the baseline assessment (N = 97), rather than all randomised participants (*N* = 124). Percentages are calculated using the available baseline sample within each allocation arm unless otherwise indicated.

### 3.4 Secondary outcomes: preliminary clinical effects

The analysis used linear mixed-effects models to assess change over time while accounting for within-participant correlation and incorporating available repeated outcome data. Clinical outcomes were examined in two stages, distinguishing the controlled between-group comparison from subsequent exploratory analyses following intervention receipt.

The principal controlled-phase analysis compared change from Baseline 1 to post-intervention in the immediate intervention group and to the end of the waiting period (Baseline 2) in the waitlist control group, before the latter commenced treatment. Although baseline outcome data were available for 97 participants, controlled-phase endpoint questionnaires were returned by 79 participants. Missing endpoint questionnaires were proportionally more frequent in the immediate intervention group (29.5%) than in the waitlist control group (9.4%).

Subsequent exploratory analyses examined outcomes following intervention receipt, irrespective of allocation timing, using available post-intervention and three-month follow-up observations. These analyses were not interpreted as controlled between-group treatment comparisons, as participants in both groups had received the intervention.

Linear mixed-effects models incorporated available repeated outcome observations rather than restricting analyses to complete cases. These analyses assumed that missing outcomes were missing at random (MAR), conditional on the variables included in the models. As this assumption cannot be verified from observed data, the findings were interpreted cautiously in light of incomplete outcome data and differential missingness. The potential implications of departures from MAR are considered in the Discussion.

#### 3.4.1 Between-group comparisons

Between-group differences at post-intervention were examined using linear mixed-effects models, with baseline-adjusted ANCOVA conducted as a sensitivity analysis. Findings were broadly consistent across both approaches (Table 3, Table 4, Fig. 3). For descriptive statistics of outcomes by group across time points see Table S4 in supplementary information.

**Table 3:** Between-group differences (immediate intervention group and waitlist control group), baseline to post-treatment.

Outcome	*N*	Immediate: pre-intervention (Baseline) *M*	Immediate: post-intervention *M*	Waitlist control baseline 1 (start of waiting period) *M*	Waitlist control baseline 2 (end of waiting period) *M*	Immediate intervention: change pre-post	Waitlist control: change during waiting period	Between-group Δ	Pooled *SD*	Cohen's *d*	95% CI	Phase × condition
PG-13-R	97	35.61	26.90	33.25	31.64	−8.72	−1.61	−7.11	3.81	−1.87	[−2.44, −1.30]	*F*(1, 80.11) = 33.36, *p* < .001
PCL-5	97	38.16	24.37	35.01	32.13	−13.79	−2.88	−10.91	7.03	−1.55	[−2.19, −0.91]	*F*(1, 79.43) = 22.59, *p* < .001
PHQ-9	96	11.34	6.89	10.98	9.57	−4.45	−1.41	−3.05	2.96	−1.03	[−1.69, −0.38]	*F*(1, 79.76) = 10.00, *p* = .002
GAD-7	96	8.64	6.49	9.58	9.20	−2.15	−0.38	−1.77	2.50	−0.71	[−1.21, −0.20]	*F*(1, 79.44) = 4.72, *p* = .033
SCS	97	2.68	3.09	2.67	2.68	0.41	0.01	0.40	0.34	1.15	[0.52, 1.77]	*F*(1, 81.03) = 12.54, *p* < .001
OG-SD	97	69.23	64.24	70.66	69.87	−4.99	−0.79	−4.20	8.63	−0.49	[−1.13, 0.16]	*F*(1, 77.71) = 2.20, *p* = .142

*Note. Values are estimated marginal means derived from linear mixed-effects models. The controlled-phase comparison examines change from the initial assessment to the controlled endpoint. For the immediate-intervention arm, this represents pre- to post-intervention change; for the waitlist control arm, it represents change from the start to the end of the waiting period, before intervention commencement. Models incorporated available repeated outcome data under a missing-at-random assumption. Sample sizes therefore reflect available data rather than complete observations at every assessment point. Models included random intercepts for participants and were estimated using restricted maximum likelihood (REML) with Satterthwaite approximation for degrees of freedom. Effect sizes (d) were calculated using pooled model residual standard deviations. Negative values indicate reductions in symptoms for PG-13-R, PCL-5, PHQ-9, GAD-7, and OG-SD; positive values indicate improvement for the Self-Compassion Scale (SCS).* Assessments collected after the Waitlist Control group subsequently received the intervention were not included in these between-group analyses and are reported separately in Table 5.

**Table 4:** Baseline-adjusted between-group differences at the controlled-phase endpoint.

Outcome	*N*	*R*^2^	Baseline *M*	Group effect (waitlist control vs. immediate intervention) *B*	*SE*	95% CI	Adjusted *M* (immediate intervention post-intervention)	Adjusted *M* (waitlist control baseline 2; pre-intervention)	*RMSE*	Cohen's *d*	*p*
PG-13-R	77	0.68	34.18	7.05	1.28	[4.54, 9.56]	25.54	32.59	5.43	1.30	< 0.001
PCL-5	76	0.69	36.00	10.42	2.26	[5.99, 14.85]	22.78	33.20	9.56	1.09	< 0.001
PHQ-9	76	0.59	10.99	2.82	0.91	[1.04, 4.61]	7.64	10.46	3.89	0.73	0.003
GAD-7	76	0.61	9.07	1.99	0.77	[0.45, 3.53]	6.74	8.73	3.26	0.61	0.012
SCS	75	0.55	2.65	−0.40	0.11	[−0.61, −0.19]	3.05	2.65	0.46	−0.86	< 0.001
OG-SD	75	0.72	69.97	4.38	2.78	[−1.08, 9.83]	65.79	70.16	11.80	0.37	0.120

*Note*. N represents the number of participants with complete baseline and controlled-endpoint data for each outcome-specific ANCOVA model. Sample sizes vary because of missing data at the individual outcome level. Models adjusted for the corresponding initial baseline score. The controlled endpoint represents post-intervention assessment in the immediate-intervention arm and assessment at the end of the waiting period, before intervention commencement, in the waitlist control arm. Positive B values indicate higher scores in the Waitlist Control group relative to the Immediate Intervention group after adjustment for baseline. For the Self-Compassion Scale (SCS), higher scores indicate improvement; therefore, negative B values reflect greater improvement in the Immediate Intervention condition. B = unstandardised regression coefficient; SE = standard error; CI = confidence interval; RMSE = root mean square error; d = standardised between-group effect size. The adjusted means differ slightly from the estimated marginal means presented in Table 3 because ANCOVA adjusts for baseline only, whereas linear mixed-effects models account for repeated observations and within-participant correlation over time.

**Fig. 3:**
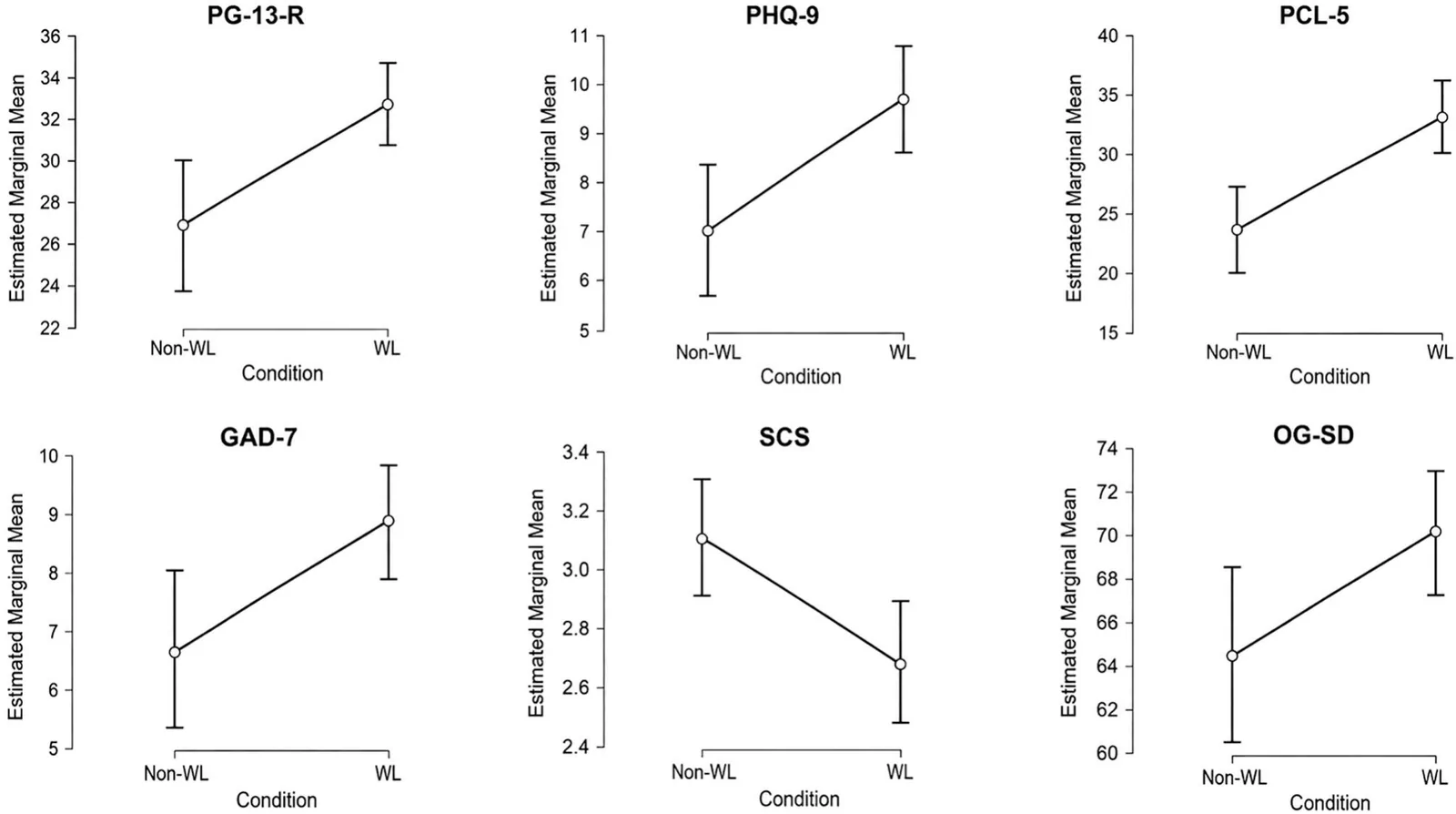
Baseline-adjusted between-group differences during intervention phase. Note. Non-WL = immediate intervention group; WL = waitlist control group. PG-13-R Prolonged Grief Disorder-13-Revised; PHQ-9 = Patient Health Questionnaire-9 (depression): PCL-5 = Post-Traumatic Stress Disorder Checklist for DSM-5; GAD-7 = Generalised Anxiety Disorder-7 (anxiety); SCS = Self-Compassion Scale; OG-SD = Overall Grief Severity and Distress. Error bars represent 1 standard error. Higher scores indicate greater symptom severity for PG-13-R, PHQ-9, PC-5, GAD-7 and OG-SD, and greater self-compassion for the SCS.

Owing to the waitlist design, between-group analyses were restricted to the controlled trial phase. Accordingly, outcomes for the immediate intervention group were compared from Baseline 1 to post-intervention, whereas outcomes for the waitlist control group were compared from Baseline 1 to Baseline 2, immediately prior to receiving the intervention. Data collected after the waitlist control group received the intervention were analysed separately as exploratory within-group outcomes and were not included in the between-group efficacy comparisons.

Compared with the waitlist control condition, the immediate intervention group showed significantly greater reductions in prolonged grief and post-traumatic stress symptoms. Large effects were observed for prolonged grief *(PG-13-R), d = −1.87, p <* *.001* and post-traumatic stress (*PCL-5), d = −1.55, p <* *.001*. Depression (PHQ-9) also showed a large effect, *d* = −1.03, *p* = .002 while anxiety outcomes (*GAD-7)* had a medium effect*,* d *= −0.71, p = .033*. Self-compassion (SCS) increased significantly in the immediate intervention group, *d = 1.15, p <* *0 0.001*, indicating a large effect. Effects for social disconnection (OG-SD) were medium and not statistically significant, *d = −0.49, p =* .142.

### 3.5 Ancillary analyses

#### 3.5.1 Within-group change

Within-group changes by group are presented in Fig. 4, with session-by-session trajectories and descriptive statistics provided in the Supplementary Materials (Tables S5 and S6). These exploratory supplementary analyses were conducted examine within-group symptom change over time and complement the primary between-group models.

**Fig. 4:**
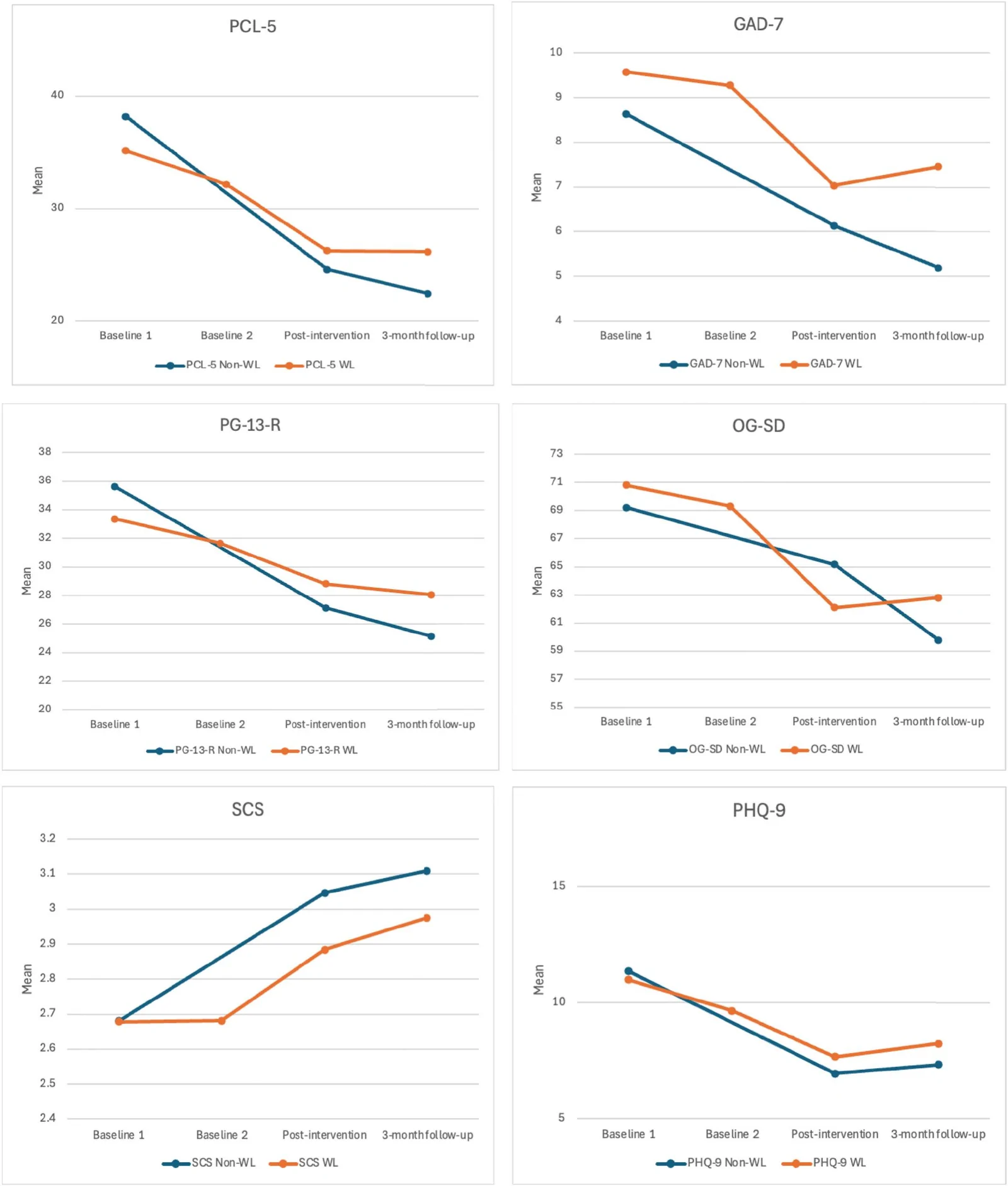
Outcome trajectories over time by group across timepoints. *Note.* Outcome trajectories are presented according to allocation arm and assessment timing. The immediate-intervention arm received treatment between Baseline 1 and the first post-intervention assessment, whereas the waitlist control arm remained untreated during this period and subsequently received the intervention following Baseline 2. Accordingly, assessments following waitlist crossover represent outcomes after delayed intervention and should not be interpreted as controlled between-group comparisons. Formal between-group treatment effects during the controlled phase are reported in Table 3, Table 4.

Analyses of weekly trajectories over the eight sessions suggested that delayed intervention access in the waitlist control group was associated with slower and more variable improvement over time for depression, prolonged grief and PTSD outcomes compared to the more consistent reductions observed in the immediate intervention group. Interpretation remains tentative given the feasibility nature of the study.

#### 3.5.2 Exploratory covariate analysis

Exploratory linear mixed-effects analyses examined whether demographic and bereavement-related covariates were associated with post-intervention outcomes across the combined sample (waitlist control and immediate intervention; *N* = 91). No consistent pattern of associations emerged across outcomes. Some exploratory interactions with time (pre- to post-intervention) were observed for gender and previous support accessed; however, these analyses were not powered for subgroup comparisons and should be interpreted cautiously. Full results are reported in Table S7 in Supplementary Materials.

#### 3.5.3 Reliable change analyses

Reliable change analyses examined the proportion of participants with clinically meaningful improvement in outcomes post-intervention and 3-month follow-up (see Table 5). These analyses complemented the inferential models by indicating individual-level change. At post-intervention, a greater proportion of participants in the immediate intervention condition demonstrated reliable improvement in prolonged grief and post-traumatic stress compared to the waitlist control group. Approximately 41.9% of participants in the immediate intervention group met criteria for reliable improvement on PG-13-R compared with 5.9% in the waitlist control group; for the PCL-5, improvement was: immediate intervention 40.0% vs waitlist control group; 3.0%. For other clinical outcomes, patterns were similar but more modest relative to the waitlist control. Participants in the immediate intervention group showed greater rates of reliable improvement in depression (PHQ-9), anxiety (GAD-7), self-compassion (SCS) and social disconnection (OG-SD). Across both groups, rates of deterioration were low across outcomes ranging from 0 to 6.7%. At 3-month follow-up, improvements in the immediate intervention group were maintained. Participants in the waitlist control group also showed delayed improvements, although these were generally smaller. Across both groups, most participants who did not show reliable improvement remained stable over time and deterioration was uncommon.

**Table 5:** Reliable change by group from baseline to post- intervention and 3-months follow up (within-group analyses).

Outcome	Time comparison	Valid n	Immediate intervention			Valid n	Waitlist (delayed intervention)		
			No reliable change, n (%)	Reliable improvement, n (%)	Reliable deterioration, n (%)		No reliable change, n (%)	Reliable improvement, n (%)	Reliable deterioration, n (%)
PGD	Pre-FU3	31	13 (41.9)	18 (58.1)	0 (0.0)	31	21 (67.7)	9 (29.0)	1 (3.2)
	Pre-Post	31	18 (58.1)	13 (41.9)	0 (0.0)	34	32 (94.1)	2 (5.9)	0 (0.0)
PCL-5	Pre-FU3	31	19 (61.3)	12 (38.7)	0 (0.0)	31	29 (93.5)	2 (6.5)	0 (0.0)
	Pre-Post	30	18 (60.0)	12 (40.0)	0 (0.0)	33	32 (97.0)	1 (3.0)	0 (0.0)
PHQ-9	Baseline-FU3	31	24 (77.4)	7 (22.6)	0 (0.0)	29	24 (82.8)	4 (13.8)	1 (3.4)
	Pre-Post	30	23 (76.7)	7 (23.3)	0 (0.0)	32	29 (90.6)	2 (6.3)	1 (3.1)
GAD-7	Pre-FU3	31	28 (90.3)	3 (9.7)	0 (0.0)	30	28 (93.3)	2 (6.7)	0 (0.0)
	Pre-Post	30	23 (76.7)	6 (20.0)	1 (3.3)	32	31 (96.9)	1 (3.1)	0 (0.0)
SCS	Pre-FU3	31	24 (77.4)	7 (22.6)	0 (0.0)	30	22 (73.3)	7 (23.3)	1 (3.3)
	Pre-Post	30	25 (83.3)	5 (16.7)	0 (0.0)	32	27 (84.4)	4 (12.5)	1 (3.1)
SD	Pre-FU3	31	24 (77.4)	7 (22.6)	0 (0.0)	31	28 (90.3)	3 (9.7)	0 (0.0)
	Pre-Post	30	23 (76.7)	5 (16.7)	2 (6.7)	32	26 (81.3)	5 (15.6)	1 (3.1)

*Note*. Reliable change analyses included participants with valid paired observations at the relevant assessment points. Pre refers to the assessment immediately preceding intervention commencement (Baseline 1 for the immediate intervention arm and Baseline 2 for the waitlist arm, following the waiting period). Post refers to the assessment immediately following intervention completion, and FU3 refers to the three-month post-intervention follow-up. Valid n represents the number of participants with paired observations for each outcome and comparison; percentages are calculated using these outcome-specific denominators. Reliable improvement and deterioration were determined using the prespecified reliable change index (RCI) thresholds. These exploratory analyses describe within-group change following receipt of the intervention and should not be interpreted as controlled between-group comparisons. PGD = prolonged grief disorder; PCL-5 = PTSD Checklist for DSM-5; PHQ-9 = Patient Health Questionnaire-9; GAD-7 = Generalised Anxiety Disorder-7; SCS = Self-Compassion Scale; SD = Social Disconnection.

#### 3.5.4 Baseline symptom severity relative to established screening thresholds

An exploratory post hoc analysis examined the proportion of participants scoring at or above the established self-report screening thresholds at baseline. Using the published prolonged grief threshold of ≥30 (Prigerson et al., 2021), 34/44 participants (77.3%) in the immediate intervention group and 35/52 (67.3%) in the waitlist control group scored above the threshold, giving an overall proportion of 71.9% (69/96). Using the recommended PCL-5 threshold of ≥33 (Weathers et al., 2013), 26/44 participants (59.1%) in the immediate intervention group and 25/52 (48.1%) in the waitlist control group screened positive for probable PTSD, giving an overall proportion of 53.1% (51/96). These classifications were based on self-report screening thresholds rather than clinician-administered diagnostic assessments and should not be interpreted as confirmed PGD or PTSD diagnoses.

#### 3.5.5 Sensitivity analyses accounting for therapeutic-group clustering

Exploratory sensitivity analyses were conducted to examine the impact of additionally including therapeutic group as a random intercept in the between-group models. The Phase × Condition interaction findings remained virtually unchanged across all six outcomes (Supplementary Table S8). However, estimated therapeutic-group variance was at or near zero for five outcomes, resulting in singular-fit warnings. Given the small number of therapeutic groups (*n* = 4), these findings should be interpreted cautiously.

## 4 Discussion

### 4.1 Interpretation and feasibility

This pilot randomised controlled trial evaluated the feasibility and acceptability of a therapist-led online group intervention for individuals bereaved by cancer and explored preliminary changes in psychological outcomes to inform a future fully powered trial. Overall, findings indicated that the intervention was feasible to deliver and acceptable to participants, with preliminary evidence of improvement across multiple psychological outcomes.

Overall, the pooled progression criteria were met, supporting the feasibility of proceeding to a definitive trial with refinements. Recruitment targets were achieved within the planned timeframe, and pooled retention and adherence met predefined progression criteria. However, these aggregate findings masked differences between allocation arms and assessment phases. Missing outcome data during the controlled phase were greater in the immediate intervention arm than in the waitlist control arm, whereas intervention discontinuation following treatment commencement was higher among participants receiving delayed intervention. These findings highlight the need to refine strategies for supporting intervention engagement and research follow-up, and to consider differential missingness in the design and analysis of a future definitive trial.

These findings are notable given the challenges commonly reported in bereavement research (Marello et al., 2026; Caserta et al., 2010) and suggest that online delivery may reduce barriers associated with geography, caregiving responsibilities and help-seeking stigma (Aoun et al., 2015). Consistent with previous research, therapist-led online bereavement interventions appear both feasible and acceptable to participants (Knowles et al., 2017; Delgado-Corcoran et al., 2024). Participants may benefit from the accessibility of digital delivery alongside opportunities for peer connection within structured therapeutic environments (Supiano et al., 2020; Drouin et al., 2025; Uneno et al., 2024; Phillips et al., 2025). The present findings extend emerging evidence that CBT-informed online bereavement groups can be successfully implemented within routine charitable services (Smith et al., 2025). They also contribute to the distinction between structured therapeutic interventions and peer-support approaches, suggesting that therapist-led groups may more directly target cognitive, behavioural and trauma-related processes associated with persistent grief reactions (Finucane et al., 2025).

### 4.2 Preliminary clinical outcomes

The secondary aim of this study was to examine preliminary changes across outcomes rather than provide definitive evidence of efficacy. Exploratory analyses suggested improvements across multiple psychological outcomes, with the largest effect sizes observed for prolonged grief, post-traumatic stress symptoms, depression and self-compassion. Medium effects were observed for anxiety, while changes in social disconnection were less consistent. These findings should, however, be interpreted cautiously given the differential missingness between allocation arms during the controlled phase and the potential for bias arising from departures from the missing-at-random assumption.

Improvements occurred during the intervention phase and were generally maintained at three-month follow-up. Reliable change analyses indicated that a substantial proportion of participants demonstrated improvement beyond measurement error, particularly for prolonged grief and post-traumatic stress symptoms. These findings suggest that the intervention may support reductions in bereavement-related psychological distress, with improvements that are sustained in the short term.

An important consideration when interpreting these findings is that participants allocated to the waitlist condition received written psychoeducation, self-guided grief resources and signposting during the waiting period. The observed between-group differences suggest potential additional benefit associated with the therapist-assisted group intervention relative to the support provided during the waiting period. However, the independent contribution of these resources to changes in psychological outcomes could not be established.

Observed effects appeared larger than those typically reported in online bereavement interventions, although estimates should be interpreted cautiously given the exploratory nature of the study (Park and Cha, 2023; Phillips et al., 2025). The pattern of findings was more comparable to therapist-led interventions for prolonged grief (Johannsen et al., 2019) and aligns with recent service-level findings suggesting that structured group interventions may target core grief- and trauma-related processes (Smith et al., 2025). This interpretation is consistent with cognitive-behavioural models of prolonged grief, which emphasise avoidance, maladaptive appraisals and difficulties integrating the loss.

Improvements in self-compassion support emerging evidence that compassion-based approaches may reduce self-criticism and facilitate engagement with painful grief-related experiences (Uneno et al., 2024). This is consistent with the intervention's emphasis on compassion-focused exercises throughout treatment. Anxiety outcomes were more variable, reflecting the heterogeneous nature of bereavement-related anxiety, including health anxiety, fears concerning the death of others and ongoing life stressors that may be less directly targeted by grief-focused interventions (Shear and Skritskaya, 2012).

Limited change in social disconnection may reflect the enduring interpersonal challenges associated with bereavement, including adjustment to changes in identity, roles and relationships (de Vries et al., 2014; Smith et al., 2025). It may also reflect the intervention's therapeutic focus, as social disconnection was not explicitly targeted within the group protocol. Although the group format offered opportunities for shared understanding and connection, these may not necessarily translate into improved relationships beyond the group. Notably, improvements in social disconnection have been reported following individual internet-delivered cognitive therapy for prolonged grief disorder (iCT-PG), which explicitly targets this process (Smith et al., 2026). Together with similarly limited change in the previous in-person group trial, this suggests that therapeutic focus, rather than delivery modality alone, may be important. Future iterations could therefore incorporate more explicit strategies to address social disconnection and support reconnection beyond the group setting.

The high proportion of participants exceeding established screening thresholds for prolonged grief and PTSD symptoms possibly indicates grief- and trauma-related symptoms at baseline These findings support the potential relevance of accessible bereavement interventions for individuals experiencing elevated psychological distress. However, screening classifications should not be interpreted as confirmed diagnoses.

### 4.3 Limitations

Several limitations should be considered when interpreting the findings. First, generalisability may be limited by the predominantly female, White and highly educated sample, high levels of previous therapeutic support, self-selected recruitment through a bereavement charity, and the requirement to engage with an online intervention. Participants may therefore have been particularly motivated or accustomed to seeking psychological support and may not represent the wider population bereaved by cancer such as men, individuals from less advantaged or minoritised backgrounds or those experiencing digital exclusion. Bereavement interventions have also been found to yield stronger effects in self-referred or clinically referred samples than in outreach-recruited populations (Currier et al., 2008). Parental bereavement was the most common loss relationship (52.6%), consistent with an evaluation of an NHS Talking Therapies prolonged grief pathway, in which parental loss was also most frequently reported (38%; Goff et al., 2025). This may reflect patterns of presentation to specialist grief services rather than the wider distribution of bereavement-related difficulties. Additionally, the minimum six-month post-loss eligibility criterion and relatively long mean time since bereavement (3.10 years) limit applicability to those earlier in bereavement.

Second, the waitlist design may have influenced the observed between-group differences through differences in expectancy, engagement, natural recovery or access to other support (Mohr et al., 2009; Wagner et al., 2020). Longer waiting periods have also been associated with poorer subsequent treatment outcomes in routine depression care, although this may not generalise directly to bereavement interventions (Van Dijk et al., 2023). Furthermore, waitlist participants received psychoeducational materials and self-guided resources, meaning the comparator was not entirely inactive and the independent contribution of these resources could not be established.

Third, the clinical analyses did not follow a conventional intention-to-treat (ITT) approach, as the analysis population comprised 97 participants with available baseline outcome data rather than all 124 randomised participants. Missing controlled-phase endpoint data were higher in the immediate intervention group (29.5% versus 9.4% among participants with baseline data), whereas withdrawal following treatment commencement was higher in the waitlist group. Likelihood-based mixed-effects models incorporated available observations under a missing-at-random (MAR) assumption, which cannot be verified. If missingness depended on unobserved outcomes, treatment-effect estimates could be biased. No formal missing-not-at-random sensitivity analysis was conducted; therefore, preliminary effect sizes may have been inflated or attenuated. Future trials should prioritise outcome collection irrespective of treatment discontinuation, where consent permits, and prespecify missing-data sensitivity analyses.

Fourth, preliminary clinical effect estimates should be interpreted cautiously. Small pilot studies and waitlist-controlled designs may produce inflated or unstable estimates, and the large observed effects for prolonged grief and PTSD should not be assumed to represent those achievable in a definitive trial. Although substantial proportions of participants exceeded established screening thresholds for prolonged grief and PTSD at baseline, these classifications were based on self-report measures rather than clinician-administered diagnostic assessments. The findings therefore cannot establish intervention effects specifically among individuals with confirmed prolonged grief or PTSD diagnoses. The definitive trial sample-size calculation was consequently based on a more conservative effect size derived from previous research rather than the observed pilot estimates.

Fifth, previous therapeutic support differed between groups at baseline (59.1% immediate intervention versus 81.1% waitlist control). Although exploratory covariate analyses identified no consistent association with outcomes, the measure did not capture the timing, duration, intensity or perceived effectiveness of previous support. Its potential influence on outcome trajectories therefore remains uncertain. Follow-up was also limited to three months post-intervention, restricting conclusions about longer-term maintenance of observed changes.

Finally, participants were nested within four therapeutic groups, sharing facilitators, peers and group processes. Although sensitivity analyses incorporating therapeutic group as an additional random intercept produced largely unchanged findings, the small number of groups limited reliable estimation of group-level variance, with singular fits observed for five outcomes. The possibility that clustering influenced the precision of treatment-effect estimates therefore cannot be excluded. Future definitive trials should account for therapeutic-group clustering in their design, sample-size calculations and analyses.

### 4.4 Implications

Evidence from this pilot study supports the feasibility and acceptability of therapist-led online group interventions for individuals bereaved by cancer. Such interventions may offer a scalable and cost-effective approach to delivering structured bereavement support within services facing increasing demand and workforce pressures. Digital delivery may facilitate timely access to support and aligns with public health models that emphasise accessible and proportionate bereavement care (Prigerson et al., 2009; Penny, 2014).

The findings support progression to a fully powered randomised controlled trial. Achievement of predefined feasibility criteria indicates that recruitment procedures, intervention delivery and outcome measures are suitable for evaluation in a larger study. Future research should establish effectiveness through longer-term follow-up, examine potential mechanisms of change, including self-compassion, social connection and grief-related cognitions, and investigate factors associated with treatment response. Qualitative analyses may further inform understanding of engagement, acceptability and group processes.

Online delivery has the potential to reduce barriers associated with geography, travel and limited availability of in-person services. However, implementation should avoid widening existing inequalities in access, particularly among individuals with limited digital literacy or technology access (NHS England, 2023). Future research should prioritise recruitment of underrepresented groups, including men, individuals from ethnically and culturally diverse backgrounds and those experiencing disenfranchised losses. Culturally responsive intervention design and targeted recruitment strategies will be important for improving equity and strengthening the generalisability of findings (Zhou et al., 2023). Co-production with service users may also enhance engagement and acceptability in digital contexts (National Institute for Health Research, 2021).

## 5 Conclusions

This pilot trial demonstrates that therapist-led online bereavement groups are feasible, acceptable and show promise for improving psychological outcomes among individuals bereaved by cancer. A fully powered trial is warranted to evaluate long-term outcomes, subgroup effects and mechanisms of change.

## Protocol and statistical analysis plan

The trial protocol including statistical analysis plan are publicly accessible via pubmed (PMID: 41749396) https://pubmed.ncbi.nlm.nih.gov/41749396/. Submitted on 4 June 2025, published 26 February 2026.

## Data sharing

Data management and monitoring has been registered and approved by the Data Protection Office (reference No Z6364106/2024/11/103 cancer research) in line with UCL's Data Protection Policy. All anonymised data generated or analysed during this study are included in this published article.

## Dissemination policy

We hope to submit the empirical paper and systematic review for publication in peer-reviewed journals (e.g., The Journal of Loss and Trauma). This paper do not reveal the identity of anyone who took part. Authorship eligibility includes any member from The Loss Foundation who participated in delivering the intervention as well as UCL research staff involved in the research process. We aim to grant full public access to any research disseminated.

## Consent for publication

The consent form outlined to participants that their information provided for this study would be published as a report and they were asked if they would like to receive a copy of it.

## Ethics approval and consent to participate

This trial was approved by the Life and Medical Sciences Research Ethics Committee (LMS REC) at University College London (UCL) in February 2025 (Project ID No: 0183). Please see the letter confirming ethics approval in Appendix A.

## Trial registration

Feasibility and Outcomes of Therapist-led Online Cancer Bereavement Groups (NCT07002424), clinicaltrials.gov, Registered 3 June 2025, retrospectively registered https://clinicaltrials.gov/study/NCT07002424.

## Funding

(1) Research and Finance Support for the Department of Clinical, Educational and Health Psychology (CEHP), Division of Psychology and Language Sciences (PALS), University College London (UCL). A total amount of £260.32 has been provided for funding. Beyond this there is no other specific role surrounding the conceptualisation, design, data collection, analysis, decision to publish, or preparation of the manuscript. (2) The Loss Foundation is supporting funding of the study design; collection, management, analysis and interpretation of data; writing of the report; and the decision to submit the report for publication.

## Declaration of competing interest

We acknowledge the potential for role-related and funding-related competing interests. One author (the principal investigator) is conducting this study as part of a postgraduate thesis at University College London; this does not constitute a conflict of interest, as postgraduate research is a standard academic pathway and appropriate supervisory oversight ensures impartiality in data analysis and reporting. A potential competing interest arises from the involvement of The Loss Foundation, which developed and delivers the intervention under evaluation and provides financial and logistical support for the research. This overlap introduces a potential perception of bias. To mitigate this, several safeguards have been implemented, including independent academic supervision, adherence to a pre-registered protocol, transparent reporting of methods and data and multiple-author verification of all analyses and interpretations.

## Data Availability

Data management and monitoring have been registered and approved by the Data Protection Office (reference No Z6364106/2024/11/103 cancer research) in line with UCL's Data Protection Policy.
