# Supplementary material for: A pilot feasibility randomised controlled trial of an online cancer bereavement group
Source: Internet Interv. 2026 Oct 31;46:101017. doi: 10.1016/j.invent.2026.101017 (PMC13744305; doi:10.1016/j.invent.2026.101017)
Supplement: Supplementary file 1 [file mmc1.docx]

# **Appendix A**

# **Ethics confirmation**

# **Appendix B**

# **The Loss Foundation Therapeutic Group Study Timeline**

| GROUP | BASELINE QS | BASELINE Qs v2 | Sx1 | Sx 2 | Sx 3 | Sx 4 | Sx 5 | Sx 6 | Sx 7 | Sx 8 | END of T QS | FU QS |
| --- | --- | --- | --- | --- | --- | --- | --- | --- | --- | --- | --- | --- |
| 1 | 17/02/25 |  | 24/02/25 | 03/03/25 | 10/03/25 | 17/03/25 | 31/03/25 | 14/04/25 | 28/04/25 | 12/05/25 | 19/05/25 | 19/08/25 |
| 2 | 17/02/25 |  | 25/02/25 | 04/03/25 | 11/03/25 | 18/03/25 | 1/04/25 | 15/04/25 | 29/04/25 | 13/05/25 | 20/05/25 | 20/08/25 |
| 3 (WL) | 17/02/25 | 12/05/25 | 20/05/25 | 27/05/25 | 03/06/25 | 10/06/25 | 24/06/25 | 08/07/25 | 22/07/25 | 05/08/25 | 12/08/25 | 12/11/25 |
| 4 (WL) | 17/02/25 | 12/05/25 | 21/05/25 | 28/05/25 | 04/06/25 | 11/06/25 | 25/06/25 | 09/07/25 | 23/07/25 | 06/08/25 | 13/08/25 | 13/11/25 |

- Baseline questionnaires for all groups go 1 week prior to group 1 commencing.
- Weekly questionnaires go 3 days prior to next group session, leaving time for a reminder the day before.
- End of Tx questionnaires to go 4 days after last session for each group.
- Follow-up questionnaires go 3 months after the final session.

# **Appendix C**

# **The Loss Foundation advertisement**

Free Online Therapy Group for Cancer Loss - 8 sessions

The Loss Foundation, supporting those bereaved by cancer

**Dear Friends,**

You may know that here at The Loss Foundation we are passionate about providing those who need some extra support during their bereavement with a place to go. Over the last few years, the Loss Foundation has been running online therapy groups for people who have lost loved ones to cancer or Covid-19. We are going to be collaborating with University College London to explore the impact of our online therapy groups for cancer loss.

**What is the research about?**

We want to understand if the 12-week online bereavement therapy group can help improve many of the difficult grief reactions that people have when they have lost someone they love to cancer.

**How can the therapy groups help me?**We have run these groups on multiple occasions, and those that took part kindly completed questionnaires for us and gave us feedback after every session. This meant we were able to learn about what was working well and what needed improving. We were delighted to learn that the groups helped reduce peoples’ grief intensity, low mood, and anxiety. We hope this is the case for you too, and that you get much more from the groups. As well as helping with difficulties, we hope the therapy groups help people make stronger connections with others in a similar situation. There’s only one way to know if it will help, and that’s to give it a go!

**How are the therapy groups different to the current support groups?**

Our current support groups are peer grief support groups, which are unstructured and based on the needs of those who come along on the day. Our therapeutic groups will be led by Clinical Psychologists/Cognitive Analytic Therapists and consist of 8 sessions over 12 weeks of structured content focusing on specific aspects of grief or other associated difficulties, for example, coping with grief, difficult memories, troubled sleep, anxiety. The sessions are aimed at helping people learn more about loss and how to cope with all that arises from it, as well as helping them make stronger connections with others in a similar situation. Every session will have a theme that will focus on trying to help you with a specific aspect of the grieving experience. We will be building on what has been shared and learned session-by-session, so attendees will need to commit to all 8 sessions.

**Who can participate in this research?**

If you have lost a loved one to cancer over six months ago, you may be eligible to join a therapy group. The groups are well suited to people who feel able to talk about their experiences in the group setting, and who feel able to engage in exercises in and between sessions.

**When will the therapeutic groups start?**

We will be running the groups from February 2025. There will be multiple groups running online starting in February and May 2025.

**Where will the group take place?**

This round of therapy groups will be taking place online.

**Can I forward this on to someone else?**

Yes, of course you can! If you know of someone who may benefit from these groups or it you work for an organisation that can forward this on to people in need, please feel free to do so.

**How do I take part or find out more?**

Dates are now up for the four online therapy groups we will be running online starting this February. You can find out more and register your interest to join using the links below. This will take you to some questions that will help us determine if you are eligible to join the group. It will take a couple of minutes to complete these questions.

24th February - Online Therapy Group for Cancer Loss (Monday group)

25th February - Online Therapy Group for Cancer Loss (Tuesday group)

20^th^ May - Online Therapy Group for Cancer Loss (Tuesday group)
21^st^ May - Online Therapy Group for Cancer Loss (Wednesday group)

**Please note that registration does not guarantee a place in the group.** Once registered, we will arrange a short telephone conversation with you in March to tell you more about the sessions to make sure they are the right fit for you and answer any questions you may have. Any data you are asked to provide will be stored securely in accordance with the Data Protection Act 2018.

With love from us all,
The Loss Foundation team, x

**Make a donation to The Loss Foundation**

**Calendar of events**

**Appendix D**

RESEARCH DEPARTMENT OF CLINICAL, EDUCATIONAL AND HEALTH PSYCHOLOGY

# **Information sheet**

**Researchers:**

**Lara King,** Trainee Clinical Psychologist at UCL, and Researcher

**Dr Kirsten Smith**, Clinical Psychologist and Head of Research at The Loss Foundation

**Dr Charlie Cole** , Clinical Psychologist and Principal Investigator at UCL

**Dr Erin Hope Thompson**, Clinical Psychologist and Head Researcher.

**Title of study: Outcomes of Online Cancer Bereavement Therapeutic Support Groups**

We would like to invite you to take part in this research project evaluating therapy groups. You should only take part in the therapy groups if you would like to, and before you decide whether you want to take part **it is important for you to read the following information** and discuss it with others if you wish. Please ask us if there is anything that is not clear, or if you would like more information.

**What is The Loss Foundation?**
The Loss foundation is a charity which provides support for **people who have lost someone they love to cancer.**

**What is the project about?**

The Loss Foundation is running eight online therapy groups (across 12 weeks) for people who have lost someone they love to cancer. The therapy groups will be facilitated by a mixture of The Loss Foundation and UCL researchers, consisting of Clinical Psychologists and High Intensity Therapists, trained in the intervention. Each session will focus on different aspects of grief that people struggle with and aims to support people in their loss and to help them manage those difficulties. We want to find out whether the groups are beneficial, and in what ways. We are also interested in people's experiences of the support group, such as what parts of it they find most and least helpful.

**Who is being invited to take part?**

We are inviting everyone who has registered an interest in the therapy groups to take part in the research. After registering interest, a short telephone screening call will be arranged to provide more information. If one or more of the following apply to you, you may not be eligible for the study:

- Adults who require the input from crisis mental health services
- Substance or alcohol misuse that would interfere with your ability to take part in the group.
- Receiving other support from The Loss Foundation (individual or support groups)
- Cannot be involved in other therapy at the same time elsewhere

**Do I have to take part?**

It is up to you to decide whether to take part. If you decide to take part you will be given this information sheet to keep and be asked to sign a consent form. Even if you do decide to take part, you are still **free to withdraw at any time without giving a reason**. Withdrawing has no consequences for your participation in any other events run by The Loss Foundation. The programme builds on material session by session and therefore full attendance is required for maximum benefit. However, we understand that last minute circumstances may get in the way of this.

**What will I be asked to do?**

If you decide to take part, you will attend all eight online sessions of support. All the group sessions will be two hours in duration and will take place from 6:30pm to 8:30pm over Zoom. None of the sessions will be recorded. We will have up to 25 people per group and two facilitators. We will ask you to complete some online questionnaires **before the sessions begin, when the sessions end and 3 months later** (these will take about **25 minutes**) as well as **before each meeting** (these will take about **10 minutes each time**). The questionnaires allow us to collect your feedback on the group and see what people are struggling with in their grief and if things change for them over time. It will ask questions around certain themes, such as mood, anxiety, grief, difficult memories and social connection.

The Loss Foundation will randomly allocate you to either the groups taking place **immediately in February or onto the waiting list for the next support groups in May**. If you are on the waiting list, we will ask you to complete the questionnaires when you sign up for the group and again before the start of your first group session. We appreciate it may feel frustrating to be on a waiting list for therapy, but we will provide details containing signposting to organisations and self-guided information on grief and common signs of wellbeing declining.

**What will happen to the information that is collected?**

You will be asked to provide personal information including name, address, age, gender, ethnicity, current psychological support and as well as your preferred contact details. The legal basis for processing your data is "public task", and any sensitive data (e.g., health information) will be processed under the condition of “scientific or historical research purposes”. Your data will be handled securely and confidentially and used only for the purposes outlined in this study. For further details on how your data will be used, stored, and your rights, please refer to the accompanying privacy notice.

**Everything that you tell us will be kept confidential**; only the research team will have access to what has been said. The only time confidentiality would be broken is if we became concerned that you or another person were at risk of serious harm. If we did need to tell someone else, we would discuss this with you first where possible and it would be managed as sensitively as possible.

Once the project is over, the results will be written up as part of a postgraduate thesis and reviewed by responsible individuals from the University. The paper may also be submitted for publication in an academic journal and used for additional or subsequent research. Reports will not reveal the identity of anyone who took part, written responses will be anonymised, and any academic publications will be shared to those who took part and on The Loss Foundation website. No other use will be made of the data without your written permission, and no one outside the project will be allowed access to the data.

**Are there any risks of taking part?**

It is possible that you could feel upset when completing the questionnaires or whilst engaging in the therapy group content, as they focus on grief. If you were to become upset, you can discuss this with the researcher or with the facilitator of the group and you will have the option of stopping. Facilitators will also send a ‘check in’ email post each session and give self-care exercises to see how are were feeling and normalise anything you found difficult. Further, signposting to suitable support options will also provided. If any unexpected results are found in relation to your health, we may contact your GP with your consent.

**What are the possible benefits of taking part?**

Our previous piloting of the groups showed that people taking part experienced a **positive impact on their grief, lower levels of depression and anxiety, and trauma symptoms**. We hope that the information we obtain from this study will advance knowledge about the principles of bereavement support groups, and improve practice to help people who join support groups in the future.

**Further information and contact details:**
If you have any questions about this study, please contact the head researcher:

Erin Hope Thompson, Clinical Psychologist - erin@thelossfoundation.org

Should you wish to make a complaint, the first point of contact will be: Dr Erin Thompson with the second point of contact being: Dr Kirsten Smith (kirsten@thelossfoundation.org). However, if you are not satisfied with how your complaint has been handled we invite you to contact the Chair of the UCL Research Ethics Committee: ethics@ucl.ac.uk

**Data Protection Privacy Notice**

For the purposes of conducting this research study, The Loss Foundation is the Data Controller. This means that any identifiable personal data will be stored on **GDPR-compliant UK based servers at The Loss Foundation with appropriate levels of security including folders on a password protected, encrypted hard-drive accessible to members of the research team.** We endeavour to minimise the processing of personal data wherever possible.

All questionnaires will be completed online via a secure link to The Loss Foundation Qualtrics, a data centre that is independently audited and data protection certified. Your responses on the online questionnaires will be made entirely **pseudonymous**, identified by a unique ID number which will be kept separately from any identifiable information on a password-protected file only accessed by the research team at The Loss Foundation. Prior to analysis, data will be fully anonymised by The Loss Foundation. This anonymous data will then be shared securely for processing by a UCL researcher. Only the research team will have access to the data. Your anonymised archived data will be stored securely and kept for a maximum of 10 years after the study has ended on GDPR-compliant storage platforms within The Loss Foundation's secure servers. Your data will not be made available to any commercial organisations but is solely the responsibility of the researcher(s) undertaking this study.

You have the right to access, rectify, or request the deletion of your data. **If you withdraw from the study, you can request to have all the data you have provided deleted, including contact details.** Please note: deletion of data is only possible up until the 1^st^ January 2026 after which the data will be fully anonymised. If you are concerned about how your personal data is being processed, or if you would like to contact us about your rights, please contact UCL in the first instance at data-protection@ucl.ac.uk.

**Thank you for reading this information sheet and for considering to take part in this research study.** This study has been approved by the Life and Medical Sciences Research Ethics Committee (LMS REC) Project ID No: 183.

This project is funded by University College London (UCL) and the Loss Foundation Charity You will be given a copy of this information sheet to keep.

**Getting support when you need it most**

The bereavement process can be an unpredictable and frightening journey. Here at The Loss Foundation we understand that sometimes things can feel out of control and hopeless. Our service provides meetings where people who have lost loved ones to cancer can get support and share their experiences of grief. It may be that you require more support outside of our remit. Therefore, we suggest contacting the following services for support if you are struggling to cope or finding things difficult.

**Helplines / Other organisations**

**Samaritans** (116 123) operates a 24-hour service available every day of the year. If you prefer to write down how you are feeling, or if you are worried about being overheard on the phone, you can email Samaritans at jo@samaritans.org

**At a Loss** is a charity that helps people find bereavement support in their local area. You can visit them at www.ataloss.org for more information.

**Cruse Bereavement Care** - (0808 808 1677) The Cruse National Helpline is staffed by trained bereavement support volunteers who offer emotional support to anyone affected by bereavement. Their volunteers are there to help you talk things through. They can also help you find your local Cruse service, or signpost you to support groups, financial advice, and other useful sources of information. The telephone helpline is open Monday-Friday 9.30-5pm, with extended hours on Tuesday, Wednesday and Thursday evenings, when they are open until 8pm.

**Macmillan Online Community** is an online forum where people share experiences, ask question, vent emotions and find others who understand. There are two bereavement chat groups that can be found at *https://community.macmillan.org.uk*

**It’s Time**

At support community for young people have experienced the death of a parent. You can visit their website at https://www.itstimecharity.co.uk/

**National Bereavement Service**

The NBS offer support regarding the practical and legal administration needed after someone has died. You can access their website via https://thenbs.org/ for thorough guidance surrounding all the practical elements that need to be considered after a loss.

**The Compassionate Friends**

The Compassionate Friends is a charitable organisation of bereaved parents, siblings and grandparents dedicated to the support and care of other similarly bereaved family members who have suffered the death of a child or children from a month old and from any cause. You can visit them at https://www.tcf.org.uk/

**Widowed and Young (WAY)**

Widowed and Young (WAY) is a national charity supporting men and women under the age of 50 when their partner dies. You can join as a member to meet others in a similar situation outlined on their website: https://www.widowedandyoung.org.uk/

We want you to get the support that you want, when you need it most. Please seek support from any of the above organisations, and let us know of any others that you have found helpful. We are unable to offer a 24-hour crisis service at this point. If you are struggling to keep yourself safe we recommend making an appointment with your GP to talk about sources of support. If you are unable to get in touch with your GP then your local **Accident and Emergency Department** will be able to make sure that you feel supported. Talking to someone can help you see beyond feelings of loneliness or despair and help you realise there are options.

**Please note we are a UK based charity and therefore signposting to other UK based organisations. If you are accessing our support from other countries we encourage you to become familiar with other services that can provide additional support locally to you.**

# **Appendix E**

RESEARCH DEPARTMENT OF CLINICAL, EDUCATIONAL AND HEALTH PSYCHOLOGY

# **Consent form**

**Outcomes of Cancer Bereavement Online Therapy Groups**

**Informed Consent Form for Participants**

**Please complete this form after you have read the Information Sheet and listened to an explanation about the research.**

**Title of study**: **Outcomes of Cancer Bereavement Therapeutic Support Groups**

**This study has been approved by the Life and Medical Sciences Research Ethics Committee (LMS REC). Project ID No: 183.**

Thank you for considering taking part in this research. The person organising the research must explain the project to you before you agree to take part. If you have any questions arising from the Information Sheet or explanation already given to you, please ask the researcher before you decide whether to join in. You will be given a copy of this Consent Form to keep and refer to at any time.

**I confirm that I understand that by ticking/initialling each box below I am consenting to this element of the study. I understand that it will be assumed that unticked/initialled boxes means that I DO NOT consent to that part of the study. I understand that by not giving consent for any one element that I may be deemed ineligible for the study.**

|  |  | Tick Box |
| --- | --- | --- |
| 1. | I confirm that I have read and understood the Information Sheet for the above study. I have had an opportunity to consider the information and ask questions which have been answered to my satisfaction. |  |
| 2. | I consent to taking part in the online bereavement group with the understanding that where possible, full attendance to the group is required. |  |
| 3. | I understand the eligibility criteria explained to me by the researcher during my screening telephone call.  I hereby confirm that: I **do not** fall under the exclusion criteria and am therefore am eligible for the study. |  |
| 4. | I understand there may be a delay in joining the online bereavement group by up to 3 months as people are randomly allocated to groups taking place in either February or May. I therefore consent to the possibility of being on the waiting list for the support group. |  |
| 5. | I understand that **my personal information** (including name, address, age, gender, ethnicity, current psychological support and preferred contact details) will be handled securely and confidentially and used only for the purposes explained to me. I understand that according to data protection legislation, my personal data will be processed on the legal basis of public task to conduct research in the public interest. Additionally, any sensitive data (such as health information) will be processed under the condition of scientific or historical research purposes.  I consent to the processing of my personal information for the purposes of this study only. |  |
| 6. | I understand that all personal information will remain **confidential** unless there are compelling and legitimate reasons for this to be breached. If this was the case, I would be informed of any decision that might limit my confidentiality being identified. |  |
| 7. | I understand that my data gathered in this study will be stored securely in accordance with the Data Protection Act 2018 and that all efforts will be made to ensure I cannot be identified. |  |
| 8. | I understand that other approved researchers will have access to my pseudonymised and eventually anonymised data. |  |
| 9. | I understand that the data will not be made available to any commercial organisations but is solely the responsibility of the researcher(s) undertaking this study. |  |
| 10. | I understand that my participation is voluntary and that I am free to **withdraw** at any time without giving a reason. |  |
| 11. | I understand that if I decide to withdraw, any personal data I have provided up to that point will be deleted unless I agree otherwise, up until 1^st^ January 2026 which is the cut-off date for withdrawal post-participation. |  |
| 12. | I understand the potential risks of participating and the support that will be available to me should I become distressed during the course of the research. |  |
| 13. | I agree that my GP may be contacted if any unexpected results are found in relation to my health. |  |
| 14. | I am aware of who I should contact if I wish to lodge a complaint. |  |
| 15. | I understand the direct/indirect benefits of participating. |  |
| 16. | I understand that the information I have submitted will be published as a report and I wish to receive a copy of it. Yes/No |  |
| 17. | I agree that my anonymised research data may be used by others for future research. |  |
| 18. | I understand that my anonymised information may be subject to review by responsible individuals from the University (to include sponsors and funders) for monitoring and audit purposes. |  |
| 19. | I would be happy for the data I provide to be archived at The Loss foundation for up to 10 years. |  |

**Signed:**

**Date**:

**Investigator's Statement**

**I……………………….**

Confirm that I have carefully explained the purpose of the study to the participant and outlined any reasonably foreseeable risks or benefits (where applicable).

**Signed:**

# **Appendix F**

# **Example of Intervention Manual**

**Week 2: Self-compassion**

| **WEEK** | **TIME** | | **ACTIVITY** | **FACILITATOR** |
| --- | --- | --- | --- | --- |
| **2** | 15 mins | 6:30-6:45 | **2.0 Recap of last week**  Last week recap  Home tasks feedback  Introduce content for session | Lisa |
|  | 25 mins | 6:45 - 7:10 | **2.1 Introduction to self-compassion**  - Backdraft  - Myths of compassion  - What do we mean by compassion? / In practice | Emma |
|  | 15 mins | 7:10 - 7:25 | **2.2 The flows of compassion and Kristen Neff exercise** | Emma |
|  | 5 mins | 7:25 - 7:30 | **BREAK** |  |
|  | 20 mins | 7:30 - 7:50 | **2.3 The model of self-compassion** | Lara |
|  | 30 mins | 7:50 - 8:20 | **2.4 Group exercise  Compassionate voice / Hand on heart exercise** | Lisa |
|  | 5 mins | 8:20 - 8:25 | **2.5 Compassionate anchor exercise** | Lara |
|  | 5 mins | 8:25 - 8:30 | **2.6 Closing** Set home tasks  Questions | Lara |

# **Appendix G**

# **Pre-specified Traffic Light Progression Criteria (Avery et al., 2017)**

Criteria were informed by recommendations for pilot and feasibility studies (Avery et al., 2017). Traffic-light ratings were determined using pooled trial estimates; arm-specific estimates were examined descriptively.

| **Feasibility Outcome** | **Subcomponent** | **Green (Go)** | **Amber (Amend)** | **Red (Stop)** | **Notes / Data Source** |
| --- | --- | --- | --- | --- | --- |
| **Recruitment / Uptake** | Referral & expression of interest | ≥70% of target | 50-69% | <50% | Number expressing interest / referred to the trial |
|  | Eligibility rate | ≥70% of screened eligible | 50-69% | <50% | % Meeting eligibility criteria |
|  | Consent rate | ≥70% of individuals consented | 50-69% | <50% | % providing formal consent |
|  | Randomisation rates | ≥70% of consented individuals randomised | 50-69% | <50% | % Randomised, allocation via online web-based system |
| **Session Adherence** | Attendance | ≥60% attend at least ≥50% of sessions | 40-59% | <40% | Zoom attendance records |
| **Data Completeness** | Outcome measures | ≥70% participants provide complete data | 50-69% | <50% | Completion of baseline, post-intervention, and 3-month follow-up measures via Qualtrics |
| **Acceptability** | Session helpfulness (HAT3 mean range) | ≥70% positive ratings (≥3.0/5) | ≥3.0  50-69% | ≥3.0 <50%  <60% | Helpful Aspects of Therapy questionnaire completed after each session |
|  | ≥1 additional helpful event reported | ≥80% report at least one | 60-79% | <60% |  |
|  | End-of-programme ratings | ≥70% positive ratings on session length, format, facilitator effectiveness, group size | 50-69% | <50% | Programme evaluation, Likert-scale items |
| **Safety / Adverse Events** | Monitoring | No serious adverse events | 1-2 minor adverse events, addressed | ≥3 adverse events or safety concerns | Programme monitoring |

# **Appendix H**

# **Measures**

**Helpful Aspects of Therapy (HAT) (Llewelyn, 1988).**

The following questions relate to your experience of the last group session - we want to know what you found both helpful and unhelpful.

- HAT 1: Of the events which occurred in the last group session, which one do you feel was the most helpful or important for you personally? Please describe what the event was in the space below. (By ‘event’ we mean anything that happened in the session. It might have been something one of the group facilitators said or did, something you said or did, or something someone else in the group said or did.)
- HAT 2 Please describe what made this one event helpful/important and what you got out of it.
- HAT 3 How helpful was this particular event (0= Not at all, 1 2 3 4 (Extremely)
- HAT 4 Did anything else particularly helpful happen during this session? Yes/No
- HAT 5 Please describe what the event was:
- HAT 6 How helpful was this particular event (0= Not at all, 1 2 3 4 (Extremely)
- HAT 7 Did anything happen during the session which might have been unhelpful? Yes/No
- HAT 8: Please describe what the event was:
- HAT 9 HAT 6 How unhelpful was this particular event (0= Not at all, 1 2 3 4 (Extremely)

**Prolonged Grief Disorder-13-Revised (PG-13-R; Prigerson et al., 2009)**

Lastly, do you have any other comments on the therapy group process?

**Oxford Grief-Social Disconnection Scale (OG-SD; Smith et al., 2020)**

This questionnaire lists different thoughts that people may have after the death of a loved one. We are interested in the way that you have been thinking, **in the last month**, in regard to the bereavement that you have experienced.

Please read each statement carefully and choose the scale below which best describes how much you AGREE or DISAGREE with the statement.

## **HAT Feedback Unhelpful Events**

**Table S3**

*Participant reported unhelpful events*

| **Week** | **Non-WL (N)** | **Non-WL % (N)** | **WL (N)** | **WL % (N)** |
| --- | --- | --- | --- | --- |
| **Week 1** | 43 | 23.26% (10) | 48 | 33.33% (16) |
| **Week 2** | 41 | 19.51% (8) | 40 | 20.00% (8) |
| **Week 3** | 34 | 8.82% (3) | 36 | 16.67% (6) |
| **Week 4** | 33 | 6.06% (2) | 37 | 10.81% (4) |
| **Week 5** | 33 | 15.15% (5) | 34 | 8.82% (3) |
| **Week 6** | 31 | 3.23% (1) | 27 | 3.70% (1) |
| **Week 7** | 29 | 10.34% (3) | 32 | 9.38% (3) |
| **Week 8** | 33 | 21.21% (7) | 33 | 9.09% (3) |

## **Supplementary Analyses**

**Table S4**

*Descriptive statistics for outcomes by group and time point (Baseline 1, Baseline 2 WL, Post-intervention and end of treatment)*

| **Outcome** | **Group** | **Baseline 1 M (SD), n** | **Baseline 2 M (SD), n** | **End of Treatment M (SD), n** | **3-Month Follow-Up M (SD), n** |
| --- | --- | --- | --- | --- | --- |
| PCL-5 (PTSD) | Non-WL | 38.16 (17.46), 44 |  | 24.57 (16.29), 30 | 22.45 (16.29), 31 |
|  | WL | 35.14 (15.76), 52 | 32.17 (17.03), 48 | 26.24 (14.30), 33 | 26.16 (14.87), 31 |
| PHQ-9 (Depression) | Non-WL | 11.34 (6.32), 44 |  | 6.93 (5.91), 30 | 7.32 (5.87), 31 |
|  | WL | 10.98 (6.24), 52 | 9.64 (6.44), 47 | 7.67 (4.85), 33 | 8.23 (4.68), 31 |
| GAD-7 (Anxiety) | Non-WL | 8.64 (5.30), 44 |  | 6.13 (5.22), 30 | 5.19 (4.81), 31 |
|  | WL | 9.58 (5.04), 52 | 9.28 (4.81), 47 | 7.03 (4.61), 33 | 7.45 (4.67), 31 |
| SCS (Self-compassion) | Non-WL | 2.68 (0.62), 44 |  | 3.05 (0.73), 30 | 3.11 (0.70), 31 |
|  | WL | 2.68 (0.64), 52 | 2.68 (0.62), 47 | 2.88 (0.69), 33 | 2.98 (0.73), 31 |
| OG-SD (Social disconnection) | Non-WL | 69.23 (21.93), 44 |  | 65.17 (22.95), 30 | 59.77 (23.78), 31 |
|  | WL | 70.81 (21.72), 52 | 69.32 (21.34), 47 | 62.09 (20.89), 33 | 62.81 (23.36), 31 |
| PG-13-R (Grief) | Non-WL | 35.61 (8.26), 44 |  | 27.13 (10.10), 31 | 25.16 (8.96), 31 |
|  | WL | 33.37 (8.03), 52 | 31.65 (8.78), 48 | 28.82 (7.96), 34 | 28.07 (9.01), 31 |

**Table S5**

***Linear Mixed Model Results Examining Weekly Change Across WL and Non-WL Groups***

| **Outcome** | **Effect** | **F(df)** | **P** | **Partial η²** | **Interpretation** |
| --- | --- | --- | --- | --- | --- |
| **SCS** | Week | 8.15 (7, 408.69) | < .001 | .123 | Significant change over time across participants |
|  | Group | 0.05 (1, 88.93) | .821 | .001 | No overall group difference |
|  | Week × Group | 1.07 (7, 408.69) | .384 | .018 | Similar self-compassion trajectories, |
| **PHQ-9** | Week | 6.40 (7, 407.92) | < .001 | .099 | Depression symptoms changed over time |
|  | Group | <.001 (1, 80.67) | .996 | .000 | No overall group difference |
|  | Week × Group | 2.08 (7, 407.92) | .044 | .034 | Group differed in symptom trajectory - |
| **GAD-7** | Week | 4.09 (7, 410.20) | < .001 | .065 | Anxiety symptoms changed over time |
|  | Group | 1.27 (1, 81.83) | .263 | .015 | No overall group difference |
|  | Week × Group | 0.63 (7, 410.20) | .735 | .011 | Similar anxiety trajectories |
| **PG-13-R** | Week | 7.00 (7,404.94) | < .001 | .108 | Grief symptoms changed over time |
|  | Group | 0.26 (1, 81.02) | .615 | .003 | No overall group difference |
|  | Week × Group | 2.67 (7, 404.94) | .010 | .044 | Differential grief symptom trajectories |
| **PCL-5** | Week | 9.46 (2,91.98) | < .001 | .171 | PTSD symptoms changed over time |
|  | Group | 0.01 (1,74.80) | .933 | .000 | No overall group difference |
|  | Week × Group | 3.82 (2, 91.98) | .026 | .077 | Differential PTSD symptom trajectories |
| Across outcomes, significant effects of time were observed, indicating overall change during the intervention period irrespective of group allocation. No significant overall group differences were identified. However, significant Week × Group interactions emerged for depression (PHQ-9), prolonged grief (PG-13-R), and PTSD symptoms (PCL-5), suggesting that delayed intervention access may have influenced the pattern and stability of symptom change across the intervention period for these outcomes. Visual inspection of the weekly trajectory plots (Figure S1), including estimated marginal means and 95% confidence intervals, further illustrated differing symptom trajectories between the WL and Non-WL groups across these measures. Partial η² values were calculated from the reported F statistics and numerator and denominator degrees of freedom using η²p = (F × df₁)/(F × df₁ + df₂). | | | | | |

## **Table S6**

## *Weekly Descriptive Statistics by Group Across the Intervention Period*

| **Week** | **Group** | **PHQ-9 M (SD), n** | **GAD-7 M (SD), n** | **PGD13-R M (SD), n** | **PCL-5 M (SD), n** | **SCS M (SD), n** |
| --- | --- | --- | --- | --- | --- | --- |
| **W1** | WL | 9.43 (6.45), 42 | 9.14 (5.94), 42 | 30.55 (11.10), 42 | 30.38 (16.12), 30 | 2.49 (0.57), 42 |
|  | Non-WL | 11.18 (5.74), 38 | 8.42 (4.72), 38 | 33.41 (9.46), 37 | 32.21 (16.29), 30 | 2.27 (0.54), 37 |
| **W2** | WL | 9.85 (6.51), 34 | 9.88 (5.22), 34 | 29.32 (10.72), 34 | Not measured due to error | 2.35 (0.55), 33 |
|  | Non-WL | 10.77 (6.22), 35 | 8.71 (5.49), 35 | 33.49 (9.74), 35 | Not measured due to error | 2.31 (0.57), 36 |
| **W3** | WL | 9.41 (5.74), 34 | 9.27 (5.85), 34 | 29.88 (9.90), 33 | Not measured due to error | 2.42 (0.79), 33 |
|  | Non-WL | 9.73 (6.44), 33 | 7.73 (5.68), 33 | 29.64 (12.24), 33 | Not measured due to error | 2.35 (0.64), 32 |
| **W4** | WL | 9.37 (6.29), 30 | 8.37 (5.59), 30 | 28.67 (10.81), 30 | 28.85 (16.94), 34 | 2.60 (0.68), 30 |
|  | Non-WL | 9.71 (6.54), 35 | 7.60 (5.61), 35 | 30.79 (11.39), 34 | 31.56 (16.29), 34 | 2.38 (0.65), 32 |
| **W5** | WL | 9.21 (4.86), 28 | 8.57 (4.64), 28 | 28.39 (10.92), 28 | 26.95 (17.08), 30 | 2.53 (0.73), 27 |
|  | Non-WL | 9.43 (7.03), 30 | 8.33 (5.89), 30 | 31.07 (10.97), 30 | 26.83 (16.29), 30 | 2.38 (0.65), 30 |
| **W6** | WL | 9.36 (5.84), 25 | 8.52 (5.75), 25 | 28.48 (9.86), 25 | 28.04 (17.31), 25 | 2.51 (0.73), 25 |
|  | Non-WL | 8.25 (6.11), 24 | 7.33 (5.31), 24 | 29.21 (12.16), 24 | 27.74 (16.29), 24 | 2.45 (0.65), 24 |
| **W7** | WL | 9.64 (6.10), 25 | 9.12 (5.88), 25 | 31.08 (10.58), 25 | 29.13 (17.30), 25 | 2.38 (0.72), 25 |
|  | Non-WL | 8.50 (6.10), 30 | 7.67 (5.59), 30 | 28.59 (10.17), 29 | 28.19 (16.29), 29 | 2.46 (0.65), 29 |
| **W8** | WL | 7.67 (4.85), 33 | 7.03 (4.61), 33 | 28.82 (7.96), 34 | 28.57 (16.72), 33 | 2.38 (0.69), 33 |
|  | Non-WL | 6.93 (5.91), 30 | 6.13 (5.22), 30 | 27.13 (10.10), 31 | 24.57 (16.29), 30 | 2.46 (0.73), 30 |

**Figures S1, A-E**

*Trajectory of within-group change over 8 weeks across outcomes*

 

**Table S7**

*Exploratory covariate analysis*

| **Outcome** | **Predictor** | **Test Statistic** | ***P*** | **Direction of Estimated Change** | **Estimated Marginal Means (95% CI) N** |
| --- | --- | --- | --- | --- | --- |
| OG-SD (Social disconnection) *(higher scores = greater isolation)* | Time × Gender | *F*(2, 62.84) = 4.96 | .010 | Estimated trajectories differed across gender groups. Females and non-binary participants showed reductions in social disconnection over time, whereas males showed increased social disconnection, although estimates for non-binary participants were uncertain due to small sample size. | Female: Pre = 69.42 (64.78, 74.07), Post = 62.91 (58.04, 67.78) Male: Pre = 60.50 (43.14, 77.86), Post = 71.63 (52.29, 90.98) Non-binary: Pre = 86.50 (56.44, 116.56), Post = 63.86 (30.35, 97.37)  N= 91 |
| SCS (Self-compassion) *(higher scores = greater self-compassion)* | Time × Previous Therapy | *F*(1, 67.87) = 5.05 | .028 | Participants without previous therapy showed greater increases in self-compassion over time compared with those with previous therapy history. | No previous therapy: Pre = 2.59 (2.34, 2.84), Post = 3.09 (2.82, 3.37) Previous therapy: Pre = 2.71 (2.55, 2.87), Post = 2.89 (2.71, 3.07)  N= 91 |
| Exploratory linear mixed-effects models examined whether demographic and bereavement-related characteristics were associated with change over time in intervention outcomes across groups. Covariates examined included age, gender, ethnicity, education, employment, religion, time since loss, presence at death, multiple losses, and previous therapy history. Separate models were fitted for each outcome and included a random intercept for participant to account for repeated observations. Models were estimated using restricted maximum likelihood (REML). Reported estimated marginal means are model-derived estimates with 95% confidence intervals. These analyses were exploratory and not powered for subgroup comparisons; findings should therefore be interpreted cautiously. **Supplementary Table S8** Sensitivity analyses examining the impact of accounting for therapeutic-group clustering on between-group differences from baseline to post-treatment. Outcome \| N \| Original model Phase × Condition, F(df) \| p \| Sensitivity model Phase × Condition, F(df) \| p \| Therapeutic-group variance (σ²) \| Singular fit; PG-13-R \| 97 \| 33.36 (1, 80.11) \| < .001 \| 33.36 (1, 80.11) \| < .001 \| 0 \| Yes; PCL-5 \| 97 \| 22.59 (1, 79.43) \| < .001 \| 22.59 (1, 79.43) \| < .001 \| 0.000 \| Yes; PHQ-9 \| 96 \| 10.00 (1, 79.76) \| .002 \| 9.96 (1, 79.81) \| .002 \| 0.728 \| No; GAD-7 \| 96 \| 4.72 (1, 79.44) \| .033 \| 4.72 (1, 79.44) \| .033 \| 0.000 \| Yes; SCS \| 97 \| 12.54 (1, 81.03) \| < .001 \| 12.54 (1, 81.03) \| < .001 \| 0.000 \| Yes; OG-SD \| 97 \| 2.20 (1, 77.71) \| .142 \| 2.20 (1, 77.71) \| .142 \| 0 \| Yes **Note.** Original analyses included participant-level random intercepts. Sensitivity analyses additionally included therapeutic group as a random intercept to explore potential clustering within the four therapeutic groups. All models retained Phase, Condition and their interaction as fixed effects and were estimated using restricted maximum likelihood (REML), with Satterthwaite approximations for degrees of freedom. The Phase × Condition interaction represents the between-condition difference in change across the two assessment points. Therapeutic-group variance estimates were at or near zero for five outcomes, resulting in singular-fit warnings. Given the small number of therapeutic groups (n = 4), these variance estimates and associated inferential statistics should be interpreted cautiously. N represents the number of participants included in each outcome analysis. | | | | | |
