# Supplementary material for: A pilot feasibility randomised controlled trial of an online cancer bereavement group
Source: Internet Interv. 2026 Oct 31;46:101017. doi: 10.1016/j.invent.2026.101017 (PMC13744305; doi:10.1016/j.invent.2026.101017)
Supplement: Supplementary file 2 [file mmc2.docx]

# **Other information - Open Science**

**Declarations**

**Data sources, if not collected yourself**: include any paid/unpaid sources of data. Baseline data was collected by members of the research team, including volunteers at The Loss Foundation. All other data was collected by me through the online platform Qualtrics.

**Software code, instrumentation development, etc**: Describe support received to write code (from human or AI), build bespoke equipment, and any other shared work. ChatGPT was used for support with writing code in RStudio as part of the meta-analysis. Rayyan (an AI-powered systematic review management platform) was used to flag conflicts between reviewers. All inclusions and exclusions were done manually.

**AI:** include any use of AI, including generative AI, not already included in the above categories where it has been used as a functional tool to assist in the process of creating the academic work. Chat GPT were used to support reporting of bias assessment, specifically for proof reading and language refinement. Infographic material summarising the study intervention was developed using ChatGPT.

**Other:** include any other material contributions to your thesis not already included in the above categories. Declare any contributions to proofreading other than standard editorial support, such as professional editing or substantial changes made by AI. Members of the research team provided contributions to proof reading and ongoing discussions helped to inform the project write up.

**Publications:** Include full references for all published work and work that has been submitted for publication that includes material shared with the thesis on which the candidate is an author.

- For each published work give a full citation including: all authors; title; journal/book (chapter); page numbers; volume; date of publication; publisher; DOI – if available.
- If manuscripts have been uploaded to a preprint server, please give full citations including DOI.
- Under each listed publication, please identify to which chapter of the thesis the publication relates.
- For multi-authored works, state your contribution and explain where this work appears in the final thesis.

King, L., Smith, K. V., Cole, C. L., & Thompson, E. H. (2026). A protocol for a pilot randomised controlled trial of an online cancer bereavement group. *Pilot and feasibility studies*, *12*(1), 46. https://doi.org/10.1186/s40814-026-01785-y

**Signature**: Name: Lara King  Date: 10/06/2026

## **Trial Registration**

This study was retrospectively registered on ClinicalTrials.gov (NCT07002424) on 3 June 2025. ‘Feasibility and Outcomes of Therapist-led Online Cancer Bereavement Groups’ https://clinicaltrials.gov/study/NCT07002424. The study protocol and statistical analysis plan were submitted for peer review on 4 June 2025, before completion of data collection and prior to data analysis, and were subsequently published All study procedures were conducted in accordance with the approved protocol. The retrospective registration reflected the timelines associated with the DClinPsy research programme and delays in the peer-review process for the protocol manuscript, rather than changes to the study design or analysis plan.

## **Protocol and Statistical Analysis Plan**

The trial protocol including statistical analysis plan are publicly accessible via pubmed (PMID: 41749396) https://pubmed.ncbi.nlm.nih.gov/41749396/

## **Data Sharing**

Data management and monitoring has been registered and approved by the Data Protection Office (**reference No Z6364106/2024/11/103 cancer research**) in line with UCL’s Data Protection Policy. All anonymised data generated or analysed during this study are included in this published article.

## **Competing Interests**

We acknowledge the potential for role-related and funding-related competing interests. One author (the principal investigator) is conducting this study as part of a postgraduate thesis at University College London; this does not constitute a conflict of interest, as postgraduate research is a standard academic pathway and appropriate supervisory oversight ensures impartiality in data analysis and reporting. A potential competing interest arises from the involvement of The Loss Foundation, which developed and delivers the intervention under evaluation and provides financial and logistical support for the research. This overlap introduces a potential perception of bias. To mitigate this, several safeguards have been implemented, including independent academic supervision, adherence to a pre-registered protocol, transparent reporting of methods and data and multiple-author verification of all analyses and interpretations.

## **Funding**

(1) PALS Research & Finance Support - Department of Clinical, Educational and Health Psychology. A total amount of £260.32 has been provided for funding. Beyond this there is no other specific role surrounding the conceptualisation, design, data collection, analysis, decision to publish, or preparation of the manuscript. (2) The Loss Foundation is supporting funding of the study design; collection, management, analysis and interpretation of data; writing of the report; and the decision to submit the report for publication.

## **Ethics Approval and Consent to**

This trial was approved by the Life and Medical Sciences Research Ethics Committee (LMS REC) at University College London (UCL) in February 2025 (Project ID No: 0183). Please see the letter confirming ethics approval in Appendix A.

## **Dissemination Policy**

We hope to submit the empirical paper and systematic review for publication in peer-reviewed journals (e.g., The Journal of Loss and Trauma). This paper do not reveal the identity of anyone who took part. Authorship eligibility includes any member from The Loss Foundation who participated in delivering the intervention as well as UCL research staff involved in the research process. We aim to grant full public access to any research disseminated.

## **Consent for publication**

The consent form outlined to participants that their information provided for this study would be published as a report and they were asked if they would like to receive a copy of it.

## **Availability of data and materials**

Data management and monitoring have been registered and approved by the Data Protection Office (reference No Z6364106/2024/11/103 cancer research) in line with UCL’s Data Protection Policy.
